# Developing culturally appropriate physical activity promotion strategies for older Chinese adults in the UK: an evidence-based, theory-based and person-based intervention development study

**DOI:** 10.1136/bmjopen-2026-117339

**Published:** 2026-09-03

**Authors:** Yang Yang, Kimberly Lazo Green, Nan Zhang, Lisa McGarrigle, Chris Todd

**Affiliations:** 1 School of Health Sciences, Faculty of Biology, Medicine and Health, The University of Manchester, Manchester, UK; 2 Manchester Academic Health Science Centre, Manchester, UK; 3 National Institute for Health and Care Research Applied Research Collaboration - Greater Manchester (NIHR ARC-GM), The University of Manchester, Manchester, UK; 4 National Institute for Health and Care Research (NIHR) Policy Research Unit in Older People and Frailty/Healthy Ageing, School of Health Sciences, Faculty of Biology, Medicine and Health, The University of Manchester, Manchester, UK; 5 Social Statistics, Cathie Marsh Institute (CMI), Manchester China Institute (MCI), Global Network for Ageing Research on China/Chinese (GNARC), School of Social Sciences, The University of Manchester, Manchester, UK; 6 Faculty of Science and Health, Atlantic Technological University, Castlebar, Ireland

**Keywords:** Exercise, Health Equity, Aging

## Abstract

**Abstract:**

**Background:**

Older Chinese adults in the UK face unique needs when engaging in physical activity (PA), yet culturally appropriate strategies to promote PA remain limited.

**Objective:**

To develop culturally appropriate PA promotion strategies for older Chinese adults living in the UK.

**Methods:**

The intervention development comprised two stages: design and optimisation. It was informed by previous research findings and input from public and community involvement and engagement. Stage 1 combined the person-based approach (PBA), which involved developing guiding principles, an intervention planning table and a logic model, with seven steps of the Behaviour Change Wheel (BCW) to identify intervention functions, behaviour change techniques (BCTs) and delivery modes to enhance older Chinese adults’ capability, opportunity and motivation for PA. Stage 2 used think-aloud interviews with 10 older Chinese adults to optimise the intervention.

**Results:**

Six intervention functions (education, persuasion, incentivisation, training, modelling and enablement), 27 BCTs and multiple delivery modes were identified in stage 1. In stage 2, based on think-aloud interviews with 10 participants, three prototypes were retained in their original form, one new element was added and three were removed. The remaining components were retained with either minor or major modifications. The final strategies included culturally tailored PA booklets, monthly workshops and social media support groups.

**Conclusions:**

By integrating evidence, theory and stakeholder perspectives, and by combining the PBA with the BCW, this study developed the first culturally appropriate PA promotion strategies for older Chinese adults in the UK. Further research is needed to evaluate their feasibility, acceptability and effectiveness.

**STRENGTHS AND LIMITATIONS OF THIS STUDY**

- Systematic integration of the person-based approach and the Behaviour Change Wheel, informed by extensive preparatory work, including two systematic reviews and a primary qualitative study.
- Identification of 27 behaviour change techniques mapped to Capability, Opportunity, Motivation-Behaviour (COM-B) to address key behavioural determinants.
- Use of multiple delivery approaches (booklets, workshops and social media) to enhance accessibility and sustained engagement.
- Cultural specificity of the intervention may limit generalisability to other populations or settings.
- The intervention has not yet been evaluated in a controlled trial, so effectiveness cannot be determined.

## Background

 According to the WHO Guidelines on Physical Activity and Sedentary Behaviour, older adults aged 65 years and above are recommended to engage in at least 150 min of moderate-intensity or 75 min of vigorous-intensity physical activity (PA) per week, along with balance and strength activities at least 2 days per week.^1^ Despite the well-documented benefits of PA such as improved mobility, reduced risk of chronic disease and better cognitive and mental well-being,^2^ physical inactivity tends to increase with age.^3 4^ This trend is particularly evident among ethnic minority groups who may face additional challenges to participating in PA in the host country, such as language and cultural barriers.^5^

Evidence suggests that PA promotion interventions, although heterogeneous in content and delivery, are generally effective in increasing participation among older adults.^6^ To enhance their relevance and impact, these interventions should adopt culturally appropriate strategies that consider the specific needs, values and perceived barriers of the target population.^7^ In this study, the term ‘culturally appropriate’ refers to an intervention tailored to older Chinese adults in the UK by incorporating cultural, social and contextual factors influencing PA behaviour, reflecting a holistic understanding of behaviour rather than treating culture as an isolated component.^8^

In the UK, the Chinese community is one of the largest ethnic minority groups and experiences significant health inequities. Older Chinese adults are more likely to be physically inactive compared with the general population.^9^ Many Chinese immigrants maintain strong cultural ties, which can shape their PA behaviours.^10^ Our earlier qualitative interviews revealed that older Chinese adults face not only general barriers such as health problems, limited availability of suitable PA programmes and low motivation, but also culture-specific challenges, including language barriers and a lack of culturally familiar social support. Participants also expressed preferences for activities rooted in Chinese culture, such as Tai Chi.^11^ As a result, interventions developed for the general older population may not be acceptable or effective for this group. Therefore, there is a clear need to develop culturally appropriate PA promotion strategies tailored to the needs and preferences of older Chinese adults in the UK.

To address this gap, a four-phase project comprising preparatory work, intervention design, optimisation and evaluation was conducted to develop culturally appropriate PA strategies for older Chinese adults in the UK, following the Medical Research Council (MRC) framework^12^ and the person-based approach (PBA).^13^

The preparatory stage comprised two systematic reviews and a qualitative study. The first review identified nine PA interventions targeting older adults from Chinese diaspora communities.^14^ None were conducted in the UK, highlighting a clear research gap. This review also identified potentially acceptable intervention features such as combining different delivery models, using culturally appropriate language and applying behaviour change techniques (BCTs) including social support and instruction on how to perform the behaviour.^15^ The second review^16^ examined barriers and facilitators to PA among older adults from Chinese diaspora communities and found that the influencing factors aligned with the Capability, Opportunity, Motivation-Behaviour (COM-B) model. Only one UK-based study was identified,^17^ which provided limited evidence as it focused primarily on general perceptions of exercise among older Chinese adults. To fill this gap, we conducted qualitative interviews with older Chinese adults in the UK.^11^ The findings showed heterogeneity in PA engagement, ranging from meeting PA guidelines to engaging only in walking or being completely sedentary. Participants reported common barriers such as health conditions and low motivation, alongside unique challenges related to cultural norms, limited social support and a lack of exercise partners.^5 18^ Culturally rooted facilitators, such as the value of family harmony, were also identified. Collectively, these findings mapped onto the COM-B model, which underpins the Behaviour Change Wheel (BCW), suggesting that the BCW provides a useful framework to guide the development of culturally appropriate PA strategies.^19^

The objective of this article is to describe the middle two stages in the development of culturally appropriate PA promotion strategies for older Chinese adults in the UK: intervention design and intervention optimisation. Guided by the MRC framework and informed by previous findings, the design process combined the PBA with the steps outlined in the BCW and was further optimised through think-aloud interviews as suggested by the PBA. The final strategies aim to enhance older Chinese adults’ capability, opportunity and motivation to engage in PA, thereby supporting improvements in their overall well-being.

### Overall view of the method

This study adopted a participatory health research approach and reported how the PBA and the BCW were incorporated to provide detailed, step-by-step guidance for intervention design and optimisation, with support from a public and community involvement and engagement (PCIE) group. The PBA emphasises the lived experiences of the target population through iterative qualitative interviews, thereby enhancing acceptability and feasibility,^13^ while the BCW offers a theoretical foundation for behaviour change through capability, opportunity and motivation, as well as guidance for specifying intervention functions, delivery modes and BCTs.^20^

**Figure 1:**
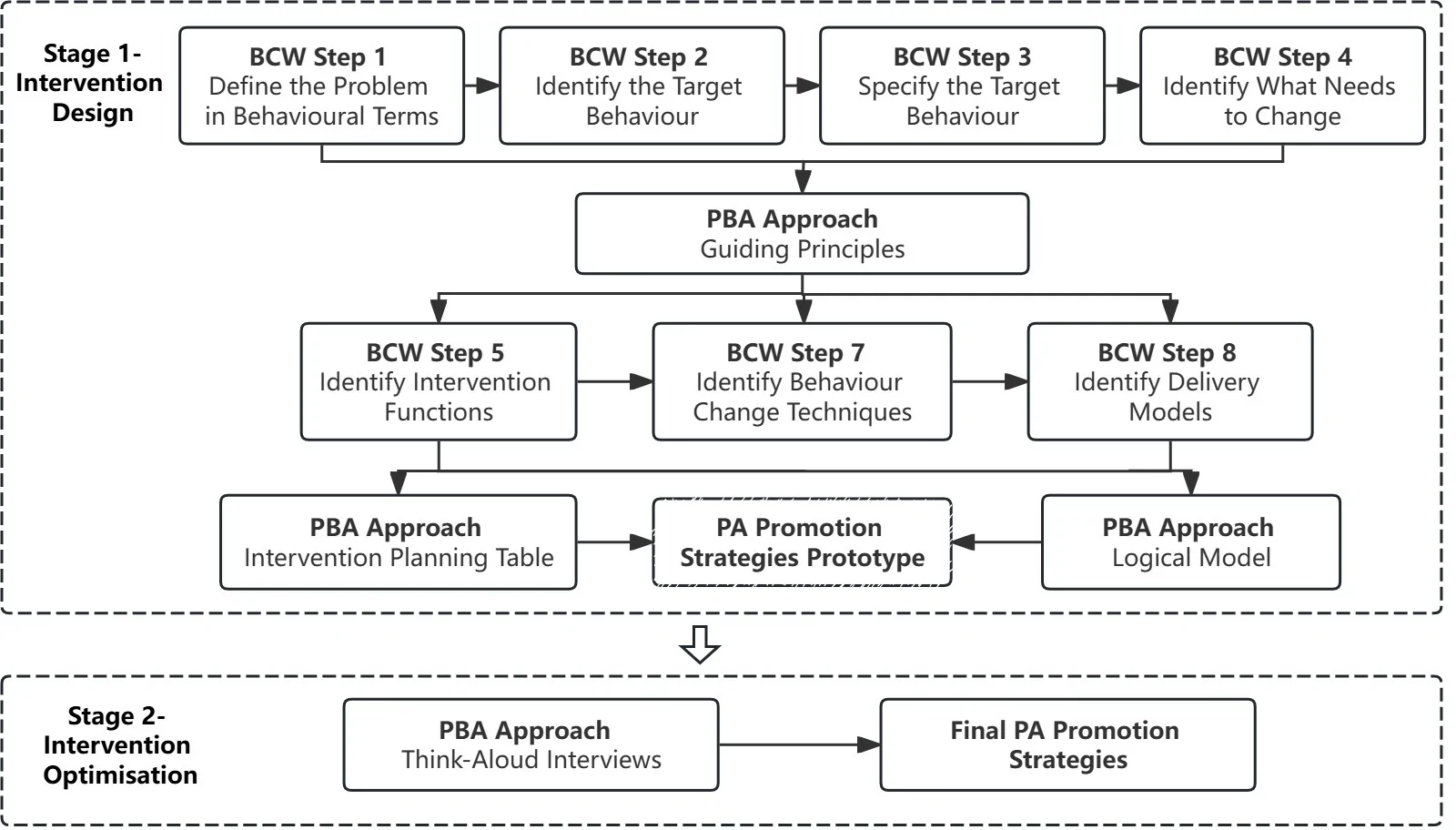
Overview of the intervention design and optimisation process guided by the BCW and PBA. BCW, Behaviour Change Wheel; PA, physical activity; PBA, person-based approach.

This paper followed the GUIDED checklist^21^ to report two key stages of intervention development (online supplemental appendix 1). Stage 1 involved intervention design, guided by the seven steps of the BCW and the PBA and stage 2 focused on intervention optimisation, conducted through think-aloud interviews as recommended by the PBA. Figure 1 illustrates the development process.

## Part 1: intervention design following the PBA and BCW

### Methods

#### Person-based approach

The PBA emphasises the value of qualitative findings and PCIE input and comprises three key stages: establishing guiding principles, developing an intervention planning table and constructing a logic model to enhance intervention acceptability.^13^

- Guiding principles were developed to ensure the intervention addressed the needs of the target population by (1) identifying barriers and facilitators to PA among older Chinese adults in the UK, (2) defining design objectives aligned with these barriers and facilitators and (3) specifying key intervention components. In this study, the guiding principles were informed by prior qualitative interviews with 27 older Chinese adults during the preparatory stage^11^ and refined with the PCIE group to ensure they reflected the most important determinants of PA behaviour.
- Intervention planning table was then constructed to synthesise evidence on barriers, facilitators, proposed features, intervention functions, BCTs, delivery modes and components.
- Logic model was created to visually map how these components are expected to promote PA and improve health outcomes among older Chinese adults in the UK.^22^

#### Behaviour Change Wheel

In addition to identifying behavioural targets for change, the BCW outlines an eight-step process for intervention development, of which seven were applied in this project.^19^ This process was led by the lead researcher, guided by the guiding principles, with regular discussions with the research team and PCIE group.

- BCW steps 1–4 focus on understanding the behaviour by defining the problem in behavioural terms, selecting the target behaviour, specifying the target population and context and analysing the behaviour using the COM-B model. These steps informed the development of the guiding principles.
- BCW steps 5, 7 and 8 focus on intervention development through the identification of intervention functions, selection of BCTs and determination of the delivery model. These steps guided the creation of the intervention planning table and the logic model. BCW step 6 (policy categories) was not applied due to limited resources and the focus on community-level and individual-level strategies rather than policy-level actions.
- APEASE criteria: The BCW recommends using the Affordability, Practicability, Effectiveness and cost-effectiveness, Acceptability, Side effects or safety and Equity (APEASE) criteria to guide the selection of intervention functions and BCTs.^19^

## Results

### BCW steps 1–4

- BCW step 1: Define the problem in behavioural terms.

Based on the findings of qualitative interviews, diverse patterns of PA were identified among older Chinese adults in the UK: (a) those meeting WHO guidelines, (b) those active but not meeting all components and (c) those largely inactive. This study targeted groups (b) and (c). In line with BCW recommendations, the problem was defined as follows: many older Chinese adults in the UK are insufficiently active and fail to meet WHO guidelines for aerobic, strength and balance activities. The behavioural definition is presented in online supplemental appendix 2.

- BCW step 2: Select the target behaviour.

Although the BCW recommends selecting a simple target behaviour to allow precise COM-B analysis, the qualitative interviews and feedback from PCIE participants led us to avoid recommending a single type of PA. Instead, participants were given the flexibility to choose activities they preferred, particularly in areas where they did not meet the guidelines, such as aerobic, balance or strength exercises. The overall goal remained to support adherence to PA recommendations.

- BCW step 3: Specify the behaviour.

After selecting the target behaviour, the BCW recommends specifying it in detail to enable accurate diagnosis. Older Chinese adults in the UK who were not meeting PA guidelines were encouraged to increase their participation in aerobic, strength and balance activities, performed regularly at suitable times, either alone or with others, to progress toward WHO recommendations. The specified behaviour is presented in online supplemental appendix 3.

- BCW step 4: Identify what needs to change.

Evidence synthesis and qualitative interviews found that factors influencing PA among older Chinese adults in the UK aligned with the COM-B dimensions.^11 16^ With PCIE support, priority factors were identified based on these findings and are listed below, with details presented in online supplemental appendix 4.

- Capability: Physical health condition; knowledge and skills related to PA.
- Opportunity: PA physical environment and programmes; PA partners; social support; cultural and language factors; and having time for PA.
- Motivation: Belief in the benefits of PA, family harmony, fear of falls and opportunities to meet people; PA as a habit, recognising its benefits and emotional influences.

### PBA-guiding principles

Based on findings from BCW steps 1–4 and informed by PCIE input, the guiding principles were developed and are presented in table 1. Older Chinese adults were categorised into three groups according to their primary barriers,^11^ providing a basis for tailored strategies targeting capability, opportunity and motivation.

**Table 1:** Guiding principles

User context	Key design objective	Key intervention components
Older Chinese adults demonstrate diverse PA behaviours and face barriers related to capability, opportunity and motivation	To understand the specific needs of individual participants and provide personalised PA promotion strategies.	Conduct a brief screening assessment to identify each participant’s needs for PA engagement, focusing on capability, opportunity and motivation.
1. Lack of capability (eg, poor health, limited knowledge and skills, language barrier, difficulties in behaviour regulation)	Modified exercises suited to physical capability are available. Increase knowledge of PA importance and benefits. Raise awareness of PA recommendations for older adults. Enhance ability to perform strength and balance exercises. Strengthen behaviour regulation and self-management skills.	Provide safe and accessible PA programmes tailored to those with physical limitations (eg, chair-based exercises). Deliver culturally relevant educational materials outlining PA benefits and WHO guidelines, integrating traditional Chinese medicine perspectives. Offer guidance and demonstrations on strength and balance exercises. Support participants in developing PA plans and self-monitoring progress.
2. Lack of opportunity (eg, limited environments, programmes, information, partners/groups, social support, time, language or cultural barriers)	Improve accessibility of PA environments and programmes. Increase awareness of available PA opportunities. Facilitate social connection and peer support. Help participants allocate time for PA. Address language and cultural barriers.	Provide information on existing PA programmes and accessible environments, especially Chinese exercise group. Facilitate opportunities to connect with PA partners or exercise groups. Offer guidance on obtaining social support. Help participants plan PA within daily routines to overcome time constraints. Present information in participants’ preferred language and culturally familiar formats.
3. Lack of motivation (eg, doubts about PA benefits; safety concerns such as concerns about falling or concerns related to conditions such as hypertension or heart disease; preference for sedentary activities)	Strengthen belief that PA benefits outweigh risks. Build confidence in managing safety concerns. Support realistic goal-setting. Encourage social engagement through PA. Promote integration of PA into daily life. Enhance enjoyment in PA participation.	Help participants identify personally meaningful PA benefits (eg, health, family harmony). Provide safety tips and reassurance to reduce concerns. Use a PA logbook for short- and long-term goal-setting and benefit tracking. Facilitate group meetings or virtual exercise sessions for mutual support. Encourage the incorporation of PA into daily routines. Create enjoyable and social PA opportunities (eg, workshops).

PA, physical activity.

- Group 1: Participants who mainly face capability barriers, including health issues, limited skills or lack of knowledge. Strategies include raising awareness of PA guidelines, teaching basic exercises and offering safe options such as chair-based activities.
- Group 2: Participants who mainly face opportunity barriers, including limited culturally appropriate environments or programmes, lack of Chinese-speaking companions and competing responsibilities. As physical environments cannot be modified, strategies focus on providing information about existing options, fostering social connections and supporting routine-based PA plans.
- Group 3: Participants who mainly face motivation barriers, including doubts about benefits, fear of injury or a preference for sedentary activities. Strategies include highlighting health benefits, providing safety tips, setting achievable goals, tracking progress and promoting social interaction and routine participation.

### BCW steps 5, 7 and 8

- BCW step 5: Identify the intervention functions.

The BCW identifies nine intervention functions: education, persuasion, incentivisation, coercion, training, restriction, environmental restructuring, modelling and enablement. Their selection was aligned with the COM-B model and guided by the APEASE criteria (online supplemental appendix 5). *Environmental restructuring* was excluded as it was not practicable or feasible due to limited resources, while *restriction* and *coercion* were deemed unacceptable as they conflict with core principles of autonomy, dignity and informed consent in healthcare and research, this position was also confirmed by the PCIE group, who emphasised the need for voluntary, respectful and non-coercive participation. The remaining functions were applied to participants within each group.

#### Group 1: lack of capability

For older Chinese adults with limited capability to engage in PA, three key intervention functions were identified: education, training and enablement. *Enablement* included low-impact options such as chair-based exercises for those in poor health and the provision of supportive equipment for home-based exercise. *Training* involved providing in-person and printed materials to teach strength and balance exercises, developing skills to set PA goals and recording PA behaviours and benefits. *Education* aimed to enhance PA knowledge based on established PA guidelines.

#### Group 2: lack of opportunity

For older Chinese adults facing barriers related to physical and social opportunity, two key intervention functions were identified: enablement and modelling. *Enablement* addressed physical opportunity barriers by providing information on accessible, culturally appropriate PA options, venues and equipment. It also targeted social opportunity barriers through workshops, social media groups and encouragement to find exercise partners. *Modelling* reinforced these strategies by promoting the sharing of PA experiences to normalise activity and foster peer support.

#### Group 3: lack of motivation

For individuals lacking reflective or automatic motivation, five BCW functions were applied: education, persuasion, incentivisation, modelling and enablement. *Education* provided information on the benefits of PA using culturally tailored content, including themes of family harmony, opportunities for social interaction and health perspectives from traditional Chinese medicine, to strengthen participants’ belief in the value of PA. It also offered safety tips to reduce fear of injury. *Persuasion* and *modelling* involved linking PA to others’ positive experiences and offering personalised encouragement from the researcher and peers. *Incentivisation* supported goal setting and reinforced progress through praise. *Enablement* helped participants develop personalised PA plans, integrate PA into daily routines, monitor progress and build sustainable habits.

- BCW step 7: Behaviour change techniques.

Based on evidence from the systematic review,^14^ the APEASE criteria, guiding principles and input from PCIE advisors, a total of 27 BCTs were identified (online supplemental appendix 6). Table 2 summarises these BCTs, with further details provided in online supplemental appendix 7.

**Table 2:** The intervention planning table

Factors	Possible intervention features	Intervention function (BCW) and BCTs	Delivery model and intervention component
**Capability** Health conditions. Lack of skills. Lack of knowledge. Behaviour regulation. Language barrier.	Provide safe and accessible PA programmes tailored to those with physical limitations (eg, chair-based exercises). Providing education on the benefits of PA. Providing education on PA recommendations. Providing instruction on PA programmes (eg, strength and balance exercises). Setting PA targets and plans. Self-monitoring PA with a logbook. Presenting information in an acceptable language.	**Intervention function:** Training; Education; Enablement **BCTs:** 1.1 Goal setting (behaviour); 1.4 Action planning; 1.5 Review behaviour goal(s); 1.6 Discrepancy between current behaviour and goal; 2.2 Feedback on behaviour; 2.3 Self-monitoring of behaviour; 4.1 Instruction on how to perform a behaviour; 6.1 Demonstration of the behaviour; 5.1 Information about health consequences; 5.6 Information about emotional consequences; 8.1 Behavioural practice and rehearsal; 9.1 Credible source	**Delivery model:** Booklet and workshop The booklet and workshops provided instructions on how to perform chair-based exercises. A booklet page introducing the benefits of PA and PA recommendations. A booklet page introducing strength and balance exercises. A workshop demonstrating strength and balance exercises and providing feedback on performance. A PA logbook enabling participants to set PA targets and plans, and to monitor their PA and achievements. All materials are available in both traditional and simplified Chinese *All information in the booklet will also be presented in the workshop.
**Opportunity** PA environment. PA programme. Information resource. PA partners. Social support. Time for PA.	Offering local PA information. Assisting participants in identifying exercise partners. Enabling participants to seek social support from family, community and healthcare professionals. Providing support to develop a feasible PA plan that can be integrated into daily life.	**Intervention function:** Enablement; Training; Modelling **BCTs:** 3.1 Social support (unspecified); 3.2 Social support (practical); 3.3 Social support (emotional); 12.2 Restructuring the social environment; 12.5 Adding objects to the environment	**Delivery model:** Booklet, social media and workshop A booklet page with information on existing PA programmes. Organising opportunities to meet in person. Creating social media groups to increase social opportunities and peer support. A booklet page with tips on integrating PA into daily life. Booklet page outlining additional support sources, including peers, family and healthcare professionals.
**Motivation** Beliefs about the positive consequences of PA. Having long-term and short-term goals. Experiencing the benefits of PA. Making PA a habit or part of the daily routine. Enjoyment of PA. Fear of falling. Preference for other activities, such as watching TV.	Introduce the benefits of PA. Identify the most relevant benefit (long-term goal). Set suitable PA targets (short-term goals). Provide safety tips to reduce concerns about potential risks. Identify a partner for peer support. Monitor and record the benefits. Provide motivational messages to increase adherence and help turn PA into a habit. Congratulate participants for any increase in PA.	**Intervention function:** Education; Persuasion; Incentivisation; Modelling; Enablement **BCTs:** 1.1 Goal setting (behaviour); 1.2 Problem-solving; 1.4 Action planning; 2.4 Self-monitoring of outcome(s) of behaviour; 5.1 Information about health consequences; 5.3 Information about social and environmental consequences; 5.4 Monitoring of emotional consequences; 6.2 Social comparison; 7.1 Prompts/cues; 8.3 Habit formation; 9.1 Credible source; 10.4 Social reward; 15.1 Verbal persuasions about capability; 16.3 Vicarious consequences	**Delivery model:** Booklet, social media and workshop A booklet page introducing the benefits of PA. A page for setting and reviewing PA targets. A booklet page outlining safety tips to reduce concerns about risk. A PA logbook page designed to help participants monitor their PA behaviour and develop a habit. A PA logbook page for tracking achievements, such as improvements in strength, balance and mood (reinforcement). Peer support through a PA partner or a social media group. Encouragement, reminders and congratulations from the researcher via the social media group.

BCT, behaviour change technique; BCW, Behaviour Change Wheel; PA, physical activity; TV, television.

- BCW step 8: Delivery model.

Based on the findings from the qualitative interviews and with input from PCIE, a flexible delivery approach was adopted instead of relying solely on a single model, which could limit accessibility or exclude certain populations (online supplemental appendix 7).

The primary delivery model consisted of a series of booklets designed to reach the majority of participants. Three monthly workshops were added to complement the booklets by delivering content in a more engaging and interactive format. These workshops provided in-person instruction on strength and balance exercises, making it easier for participants to learn, ask questions, share experiences and build motivation. They also helped foster social connections and peer support, preparing participants to join the social media group. The social media group was established to facilitate daily peer interaction and provide ongoing support from the research team.

### PBA-the intervention planning table and logic model

**Figure 2:**
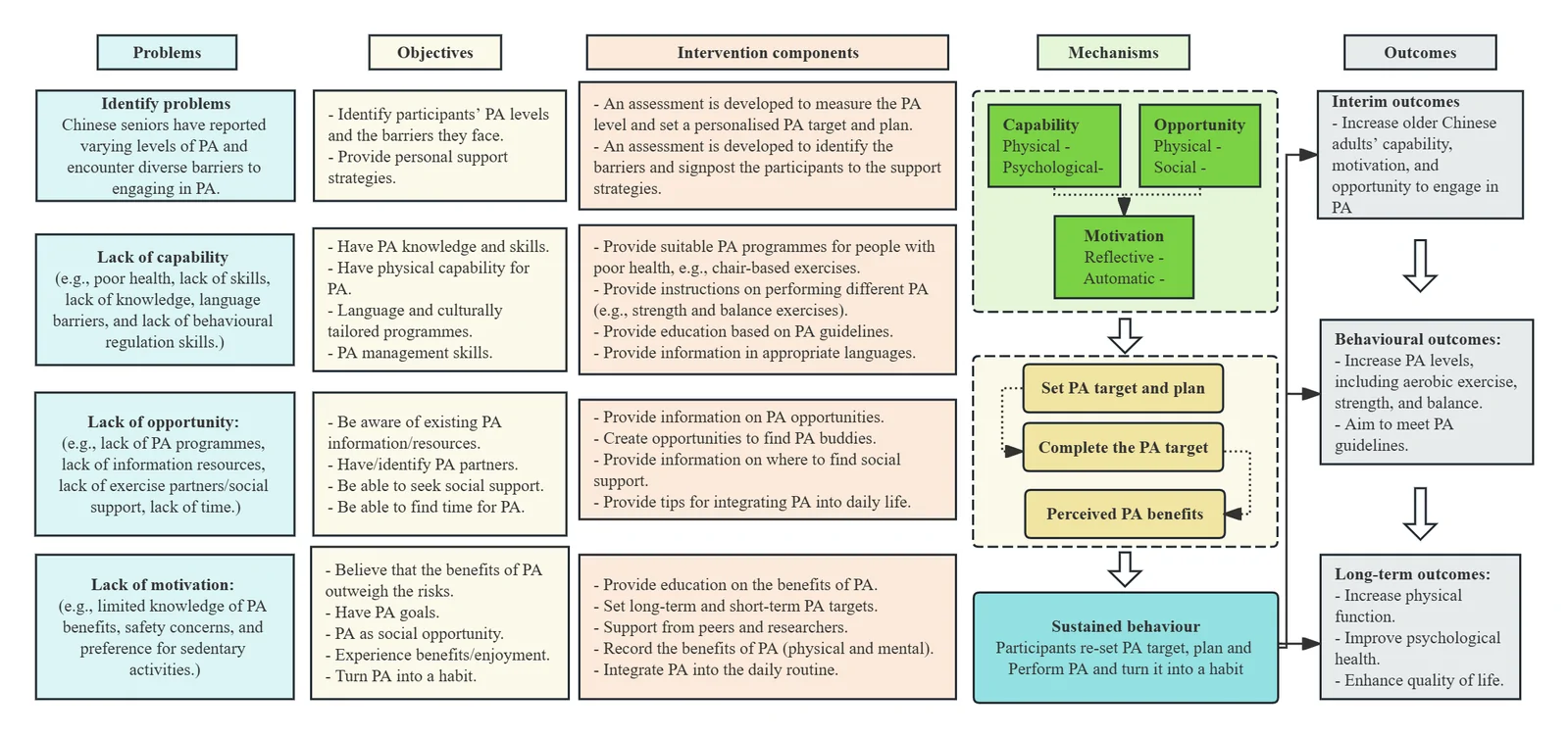
The logic model of the PA promotion strategies. PA, physical activity.

Based on the guiding principles and the findings from stages 5, 7 and 8 of the BCW, an intervention planning table was developed to map the behavioural diagnosis, selected intervention functions, BCTs and corresponding delivery methods. This table is presented in table 2. In addition, a logic model was constructed to illustrate the relationships between identified barriers, intervention components, mechanisms of action and expected outcomes. This model provides a visual summary of how the intervention is intended to bring about change and is shown in figure 2.

## Part 2: intervention optimisation through think-aloud interview

### Methods

As suggested by the PBA, think-aloud interviews enabled researchers to capture how potential users responded to the intervention prototype.^13 23^ Older Chinese adults who provided consent were invited to review the prototype and verbalised their thoughts, providing insights that informed refinements to the strategies.

In this study, think-aloud interviews were conducted in two rounds, combining concurrent and retrospective methods.^24^ In Round 1, during the initial prototype design, concurrent think-aloud interviews were conducted as the lead researcher presented potential intervention elements and collected feedback simultaneously. In Round 2, the prototype was refined into a preliminary booklet, a social media group, and a workshop (the latter two added based on Round 1 feedback). Participants used the booklet for 1 week, after which interviews were conducted to finalise the intervention optimisation.

#### Participants and recruitment

Eligible participants were aged 60 or above, self-identified as Chinese, spoke Cantonese, Mandarin or English, and were settled residents in the UK with the capacity to consent. Recruitment was conducted through Chinese community organisations and social media. Interested individuals received a participant information sheet and consent form, were given at least 3 days to decide and provided written consent before interviews were scheduled.

#### Sample size

It has been suggested that five think-aloud interviews with end-users can identify the majority of usability issues.^25^ This think-aloud study aimed to recruit a minimum of 5–10 participants, considering the financial and time constraints of the research project. Data adequacy was defined as the point at which no further intervention-related issues or substantial changes were identified through additional interviews.

#### Data collection

As suggested by the PBA, semi-structured interviews were used to collect data. Interviews were conducted in Cantonese, Mandarin or English, depending on the participant’s preference and recorded. Participants were asked about their overall views of the PA promotion programme and specific features. Probing questions were used to encourage clarification and elaboration for a deeper understanding of their perspectives. Interviews were audio-recorded with participants’ consent and conducted either face-to-face or online depending on participants’ preferences. Field notes were also taken during the interviews. The topic guide of the think-aloud interview is presented in online supplemental appendix 8.

#### Data analysis

Audio recordings were transcribed in the original interview language and analysed in NVivo by the lead researcher (YY). A rapid, iterative approach specifically recommended for think-aloud interviews was followed, with feedback tabulated to capture positive and negative comments, barriers and suggested improvements.^26^ The coding framework suggested by the PBA is presented in online supplemental appendix 9. The MoSCoW prioritisation criteria (Must have, Should have, Could have, Would like) were applied to guide modification decisions. Proposed changes were discussed with the PCIE group and research team to reach a consensus.

## Results

### Participants

A total of 10 participants were interviewed (mean age=68.2 years, range: 60–75). The majority were female (9/10; 90%), Cantonese-speaking (7/10; 70%) and all married (10/10; 100%). On average, participants had lived in the UK for 27.45 years (range: 2–45 years). Education ranged from junior high to university level. Half of the participants exercised more than four times per week, while two engaged in PA less than once a week. Participant characteristics are provided in online supplemental appendix 10.

### Findings of the think-aloud interviews

The initial PA promotion prototype, based on Part 1 findings, is presented in online supplemental appendix 11 and included a 10-page core booklet with a PA logbook, material incentives, telephone support, a WeChat group, an exercise app and a fitness tracker.

Revisions were made based on participants’ feedback. Three prototypes were retained in their original form, one new element was added and three were removed. The remaining components were kept with either minor or major modifications. Supporting quotes are presented in online supplemental appendix 12.

The final PA promotion strategies included an 8-page core booklet, a Chinese-language Otago exercise booklet,^27^ a PA logbook, 3 monthly workshops, a social media support group and an age-friendly pedometer. The details of the final intervention are reported using the Template for Intervention Description and Replication checklist,^28^ which is presented in online supplemental appendix 13.

#### Elements with no change

Three elements of the intervention were well-received and required no modifications: the *Safety Tips Page, the Story Page and the Additional Support Page* were retained in their original format, as participants found them useful and accessible.

#### Elements with some changes

Minor or significant changes were made to nine elements to improve comprehension and engagement. These included five pages of the culturally appropriate PA promotion booklet, the Chinese version of the Otago exercise booklet, the PA logbook, the social media support group and the fitness band used for PA monitoring.

The *PA Benefits Page* was simplified by replacing the image with a clearer text-based explanation, addressing concerns about clarity. In addition, participants showed positive feedback about explaining the benefit of PA from the traditional Chinese medicine view.

Two changes were made to the *PA Recommendation Page*. First, the text description was replaced with an image from the UK Chief Medical Officers’ PA Guidelines (2019).^29^ Second, culturally relevant activities such as Tai Chi and group-based dancing and singing-based social activities were added to make the content more applicable to the target audience.

To streamline the intervention content, the *Goal Setting Page* was simplified and merged with the problem identification page, allowing participants to record both their current and target PA levels in a single location. Additionally, the *Identify Problem Page* was redesigned with simpler and more concise questions to facilitate ease of use. The logic behind this design is to identify the gap between current PA levels and recommended guidelines, set achievable targets and recognise potential barriers along with support strategies.

Minor changes were made to the *Tips on Integrating PA into Daily Life Page* to remove redundancy and improve clarity. While no structural changes were made to the *PA Programme Information Page*, additional local programme information was added to enhance its relevance.

The context of the *Strength and Balance Exercise Page* was revised from simple exercises sourced from the National Health Service (NHS) website to the Chinese version of the evidence-based *Otago Strength and Balance Exercises*,^27^ as participants emphasised that the exercises should be well-designed. The Otago exercise programme was selected as an evidence-based strength and balance intervention, with practical delivery supported by a Chinese-speaking physiotherapist to enhance accessibility and acceptability for the target population.

In addition, the booklet was updated to improve readability by increasing the font size. However, the character images remained unchanged due to time and resource constraints, even though participants suggested using images of Asian women.

To improve accessibility, the *Social Media Support Group* was extended to offer both WeChat and WhatsApp options instead of using WeChat only, allowing participants to choose their preferred communication platform.

Lastly, the *Fitness Band* was replaced with an age-friendly pedometer, featuring a large display, memory function and longer battery life to better suit older adults’ needs.

#### New additions

Based on the guiding principles, the intervention aimed to provide opportunities for social contact and peer support. The original plan was to create a social media group to help participants find a virtual exercise buddy. However, participant feedback led to a major change: adding *In-person Workshops*.

Older adults disliked reading and preferred a presentation for information delivery. They felt a workshop could boost motivation and help them connect with exercise buddies. They also considered a booklet insufficient for learning the Otago exercises, preferring a professional trainer who could demonstrate movements and give real-time feedback.

In response, a workshop day was added with three parts: a presentation to introduce the booklet, a group discussion to share PA experiences and a strength and balance demonstration by a Chinese-speaking physiotherapist. Alongside the Otago group exercise, participants received a resistance band to enable them to practise strength exercises at home. These additions were designed to enhance motivation, peer support and exercise confidence.

#### Elements removed

Three components were removed due to limited feasibility or lack of participant engagement. *Material Incentives* were excluded, as participants, particularly those who were financially stable, perceived such rewards as having limited impact on behaviour, with any effects unlikely to be sustained if reliant on external incentives. *Telephone Support* was also removed, as participants were often unresponsive to calls and reported listening barriers, rendering this method ineffective for monitoring PA engagement; this was further confirmed by a PCIE member with experience in a telephone-based programme. Finally, *NHS-endorsed PA Management Apps* were not recommended, as many participants lacked access to smartphones or found the apps overly complex and language-inaccessible, limiting their usability and acceptability in this population.

## Discussion

This is the first study to develop culturally appropriate PA promotion strategies for older Chinese adults in the UK. By integrating the PBA with the BCW and drawing on findings from systematic reviews and a primary qualitative study, we identified 6 intervention functions and 27 BCTs aimed at enhancing capability, opportunity and motivation to engage in PA. Based on feedback from think-aloud interviews, the final strategies were delivered through a series of booklets, monthly in-person workshops and a social media group.

The PBA was originally developed for designing digital interventions^13^; however, it has since been applied more broadly to hybrid and offline interventions across a range of contexts in the early development phases.^30^ While the PBA enhances cultural relevance and acceptability, using it alone provides limited theoretical depth and generalisability and its reliance on qualitative insights may introduce subjectivity. The BCW addresses these limitations by offering a systematic, theory-driven framework for specifying intervention components and it has been used to develop PA interventions.^31 32^ Increasingly, the integration of the PBA and BCW has been applied in health behaviour change research to enhance both the rigour and the relevance of interventions: the PBA grounds interventions in lived experience, while the BCW ensures theoretical coherence and scalability. For example, this combined approach has been applied in digital health interventions, including hypertension self-management^31^ and cancer survivorship.^32^ Together, these studies demonstrate how combining theory-driven frameworks with user-centred design can produce context-appropriate interventions that better meet the needs of diverse populations.

Importantly, ‘culturally appropriate’ in this study does not refer solely to culture-specific components (eg, language or cultural values). Instead, it reflects a broader, population-informed approach that incorporates structural factors (eg, accessibility), social influences (eg, peer support) and sense of belonging. This is supported by evidence generated across the intervention development process, including qualitative interviews with older Chinese adults in the UK, which consistently identified that PA behaviour is shaped by structural, contextual, cultural and individual-level factors.

This broader conceptualisation is also consistent with previous guidance on developing behaviour change interventions for ethnic minority groups,^33–35^ which emphasises integrating cultural relevance with wider social, environmental and structural determinants to enhance engagement and accessibility. The intervention in this study incorporated culturally relevant materials, family and community involvement and digital support to enhance engagement and address social and environmental barriers.

In line with this, our study applied the BCW to map 27 BCTs onto behavioural drivers identified through qualitative interviews.^11^ The BCTs were selected not only through qualitative interviews and theoretical mapping but also based on evidence of commonly used BCTs reported in promoting PA among both the Chinese diaspora^14^ and the wider ageing population. For example, goal setting, action planning and the use of credible sources have been linked to increased PA engagement in older adults.^36^ Furthermore, a meta-analysis identified demonstration of behaviour, behavioural practice/rehearsal, graded tasks and instruction on how to perform the behaviour as predictors of intervention success at both post-intervention and follow-up.^37^

These BCTs were delivered through three interconnected components: a series of culturally tailored booklets, monthly in-person workshops and social media support groups. The booklets provided foundational knowledge on PA benefits, UK guidelines, safety tips, goal-setting strategies and peer stories, thereby addressing capability, opportunity and motivation. Although booklets have been widely used in health research, our PCIE group suggested that booklets alone may reduce acceptability, particularly for participants with reading barriers or low literacy. To address this, in-person workshops, introduced based on think-aloud interviews, offered a more direct and acceptable way to deliver information, while also providing opportunities for social interaction and practice through live demonstrations and peer discussions. Evidence further suggests that combining printed materials with workshops can increase PA among older Chinese adults in the USA.^38^ As a novel component, the social media support group was delivered via platforms familiar to the participants (WeChat or WhatsApp). It addressed geographical dispersion and social isolation, which are common challenges for older Chinese adults in the UK, while also supporting social motivation through prompts, peer encouragement and real-time interaction.^39 40^ However, its delivery via digital platforms may be constrained by variability in participants’ digital access and literacy, and therefore its feasibility and acceptability require further evaluation in the subsequent feasibility study. Three initially proposed components, including material incentives, telephone support and recommendations to use NHS-endorsed PA apps, were removed based on feasibility and participant feedback. The decision to exclude financial incentives was informed by think-aloud interview feedback, which suggested they were unlikely to influence behaviour among older adults with long-standing physical inactivity. This view is consistent with previous research showing mixed evidence on the effectiveness and acceptability of financial incentives for promoting behaviour change in older adults,^41 42^ and evidence indicating that any benefits may not be sustained once incentives are withdrawn. Although telephone-based interventions have shown effectiveness in adults,^43^ evidence for older ethnic minority populations is limited because barriers such as language difficulties may reduce their effectiveness. PCIE feedback also indicated that telephone support was time and resource-intensive, making it impractical for this project. It was therefore replaced with social media messaging and regular in-person meetings, though this approach may still warrant exploration in future studies if resources allow. Although the social media group component was retained, the strategy to download a new app was removed. Many participants reported low digital literacy and limited interest in health-related apps. This aligns with previous findings that older adults, particularly those from ethnic minority backgrounds, face barriers to adopting digital health tools, especially when apps are available only in English.^44 45^ Removing these elements helped tailor the intervention to the preferences and practical needs of the target population.

### Strengths and limitations

A key strength of this study is its integration of theory-driven and person-centred approaches, based on extensive preparatory work, including two systematic reviews and one primary qualitative study, ensuring that the intervention was both evidence-based and culturally relevant. The use of 27 BCTs informed by the BCW and tailored through the PBA addressed behavioural determinants across the COM-B domains. Its multimodal delivery, combining booklets, workshops and social media, enhanced accessibility and maintained social connection and motivation beyond face-to-face sessions.

However, some limitations should be noted. The intervention has not yet been tested in a controlled trial, limiting conclusions about its effectiveness. Its cultural tailoring to older Chinese adults in the UK may reduce generalisability to other ethnic groups or settings without further adaptation. The predominance of female participants in the intervention development process may have influenced the findings and intervention preferences identified, potentially limiting the transferability of the intervention to older Chinese men. A potential mismatch between participant characteristics and the intervention was identified, as the Otago exercise programme may not have been sufficiently challenging for more active individuals. Future research should therefore consider incorporating progressive exercise options and more flexible, capacity-based approaches to better match participant ability levels. The inclusion of 27 BCTs, while comprehensive, introduces complexity that may affect implementation fidelity in real-world settings. Furthermore, variations in digital literacy among older adults may impact access to and engagement with the social media component.

### Further work

Future research should evaluate the intervention in a feasibility randomised controlled trial (RCT) to establish its feasibility, acceptability and identify areas for improvement. Following this, a full-scale RCT will be required to robustly assess its effectiveness in changing PA behaviour. Consideration should also be given to implementation studies to explore how the intervention can be integrated into practice. Adaptations may be needed for delivery with other ethnic groups or in different cultural and healthcare settings to enhance generalisability and scalability. In addition, as this study did not include BCW step 6 policy-level strategies, future research should consider engaging local policymakers and community organisations to support wider implementation, scalability and long-term sustainability of the intervention beyond individual-level and community-level delivery. Moreover, future implementation may involve collaboration with community organisations, Chinese community groups and local health or social care services supporting older adults in the UK. Ongoing engagement through existing community networks and the PCIE group may help maintain relevance and acceptability, while future scalability and sustainability may require support from public health initiatives, NHS/community partnerships or future implementation funding.

## Conclusion

This study developed a culturally appropriate, theory-informed intervention to promote PA among older Chinese adults living in the UK. By integrating the BCW and PBA and delivering 27 BCTs through a multimodal model, the intervention targeted key behavioural determinants of capability, opportunity and motivation while aligning with the cultural needs of the population. Further research is needed to evaluate its feasibility, acceptability and effectiveness in supporting sustained PA in this underserved group.

### Patient and public involvement

A PCIE group comprising three older Chinese adults, one Chinese-speaking physiotherapist and one retired Chinese-speaking general practitioner contributed to the development of the intervention. They reviewed materials, advised on cultural appropriateness, supported recruitment and helped interpret think-aloud interview findings. Their feedback informed revisions to improve clarity, acceptability and relevance.

## Supplementary material

10.1136/bmjopen-2026-117339 online supplemental appendix 1

## Data Availability

Data are available upon reasonable request.
