# Supplementary material for: Developing culturally appropriate physical activity promotion strategies for older Chinese adults in the UK: an evidence-based, theory-based and person-based intervention development study
Source: BMJ Open. 2026 Sep 3;16(9):e117339. doi: 10.1136/bmjopen-2026-117339 (PMC13548221; doi:10.1136/bmjopen-2026-117339)
Supplement: Supplementary file 1 [file bmjopen-2026-117339.reviewer_comments.pdf]

PEER REVIEW HISTORY

BMJ Open publishes all reviews undertaken for accepted manuscripts. Reviewers are asked to complete a checklist review form and are provided with free text boxes to elaborate on their assessment. These free text comments are reproduced below.

ARTICLE DETAILS

Title (Provisional)

Developing Culturally Appropriate Physical Activity Promotion Strategies for Older Chinese Adults in the UK: An Evidence-, Theory- and Person-Based Intervention Development Study

Authors

Yang, Yang; Lazo Green, Kimberly; Zhang, Nan; Mcgarrigle, Lisa; Todd, Chris

---

VERSION 1 - REVIEW

**Reviewer**	**1**
**Name**	**Jansen, Carl-Philipp**
**Affiliation**	**Heidelberg University, Geriatric Center**
**Date**	**27-Apr-2026**
**COI**	**None**

Cultural adaptation: to really understand what is meant by this, clarification and definition is required. In the current stay, this is a very vague term and it is not clear what would fall under “culture”.

The BCW was applied, helping to identify 27 appropriate BCTs for this purpose. The logic model helps to illustrate the relationships between identified barriers, intervention components, mechanisms of action, and expected outcomes. The subsequent think-aloud interviews were an important step in reaching an understanding of the intevention’s potential in reaching its target. The only practical strategies that actually addressed needs specific to Chinese older adults (opposed to the non-Chinese general population) were 7, 9, and 10 (translation); the other strategies would most probably be useful to all older adults. It seems this study came with a huge effort to simply come up with three rather intuitive solutions to “make the intervention more Chinese”. I suggest to address this.

Coming out of this theory-driven approach and person-centered efforts, the intervention went through an iteration of changes that helped to sharpen its components. It will be interesting to see the results of the feasibility testing.

Why are coercion and restriction deemed unacceptable and by whom?

There is no clear reasoning why the Otago program was chosen. Is Otago appropriate? Is it ensured that this is the right fit for this population?

There are predominantly women for the intervention development (one man). Please discuss implications.

It is argued that people had limited access to smartphones for apps, but WeChat was kept in as part of the intervention despite having the same problem? Please explain.

Singing is suggested but it is not PA; please discuss.

Reviewer 2

Name Philippot, Arnaud

Affiliation KU Leuven, Research Group for Adapted Physical Activity and Psychomotor Rehabilitation

Date 28-Apr-2026

COI None

Thank you for the opportunity to review your important and innovative work. I read your manuscript with great interest, particularly your integration of the Person-Based Approach (PBA) and the Behaviour Change Wheel (BCW) to address the unique needs of older Chinese adults in the UK. Combining these frameworks is a significant challenge, and I commend the authors for their thoughtful and inspiring approach.

Below, I made some suggestions and questions aimed at further strengthening the clarity, rigor, and impact of your study. When there are several suggestion, I also choose minor revisions as I am confident the authors will address these points effectively.

Part 1.

• You mention the participation with older Chinese adults. Could you please provide more detail on the methodology employed? For instance, was a systematic approach used for data collection and qualitaitve analysis? Were interviews audio-recorded, and were field notes taken? Clarifying these aspects would enhance the transparency and reproducibility of your methods.

• Guiding Principles: The development of guiding principles is a key strength of your study. However, it is not entirely clear which elements were informed by the literature, previous qualitative studies, or direct input from the target population. Could you specify how participant input was integrated into the design objectives and intervention components? If qualitative analysis was conducted, a brief description of the process would be valuable.

• Representation of Participant Voices: To ensure the voices of participants are accurately represented, did you use a specific model or framework for qualitative analysis? If so, please consider including this information.

Table 1: Barriers and Facilitators

• Safety Concerns: Under “lack of motivation,” the phrase “build confidence in managing safety concerns” could be clarified. For example, does this refer to fears of falling, moving in

public, or other specific concerns? A brief elaboration would help readers understand the context.

Results

APEASE Criteria

Selection of Intervention Functions and BCTs: The use of APEASE criteria for selecting intervention functions and BCTs is well-justified. However, it would be helpful to explain the specific criteria used to exclude certain components. Including this information in the supplementary data would maximize transparency and allow readers to fully appreciate the decision-making process.

Delivery Model

Step 8 of BCW It's not clear if at the stage you have included the participation of the participants, could you clearly indicated when you included voices in this study of older Chinese participants living in UK?

Largely, could you indicate in each step of CBW if the opinions of your participants have been included or it was based on literature with research expert analysis.

Part 2 :

Participatory Health Research

Terminology: Consider using the term "participatory health research" instead of or in addition to "patient-centered approach," as your study actively involved participants in the research process. This would more accurately reflect the collaborative nature of your work in the research process.

Traditionally, qualitative research recommends a sample size of 12 participants for qualitative interviews to ensure methodological rigor and data saturation. While I acknowledge that budgetary constraints may have limited participant recruitment, the final sample of ten individuals could be perceived as slightly underpowered. To further strengthen the methodological justification and be more aligned with the participatory research I encourage the authors to cite relevant literature supporting the adequacy of this sample size in participatory research required ; 9-12 participants, corresponding to your study with the inclusion of 10 participants. For example, recent studies in this field have successfully employed similar sample sizes, as demonstrated in:

https://doi.org/10.1016/j.mhpa.2026.100758

https://formative.jmir.org/2024/1/e51693

Workshop Details: Could you provide more information on how the workshops were conducted? For example, what activities were included, and how were they structured to engage participants?

Clarification of PBA and BCW Integration: If audio recordings and qualitative analysis were used to inform the selection of BCTs, consider including this information in Stage 1 to highlight the participatory nature of your approach.

Cultural Appropriateness

Your introduction effectively highlights the importance of culturally appropriate interventions. In the discussion, consider further emphasizing how your intervention's cultural components (e.g., booklets, workshops, social media groups) address the specific needs of older Chinese adults. Comparing your findings with similar studies would provide valuable context and support for your conclusions, such as:

https://link.springer.com/article/10.1007/s10903-024-01587-5

https://www.sciencedirect.com/science/article/pii/S0165032725007001?casa_token=4pDZbnqJcvsAAAAA:CLMvvaIXHXkTWs45QJyKyt-p_iTfxYeF_dRaLm6R8btXUQ8Fu7FyqvthzpQuzFgEC7fCWq2IygQ

Policy Categories (BCW Step 6), while the focus on community- and individual-level strategies is understandable, the exclusion of policy-level actions in step 6 due to resource constraints is noted. Could you discuss how future research might address this gap, perhaps by exploring partnerships with policymakers or community organizations to enhance the scalability and sustainability of your intervention?

To ensure the sustained and scalable impact of your intervention, it is essential to clarify which stakeholders will be responsible for its ongoing delivery, implementation, and updates. Could the authors elaborate on the following points?

• Identification of Stakeholders: Who are the key individuals or organizations envisioned to lead and maintain the intervention ?

• Engagement Strategies: How do you plan to engage these stakeholders and participants in the long term? Are there existing partnerships or networks that could facilitate their involvement?

• Funding and Resources: What mechanisms or funding sources will support the continued operation of the intervention?

If not, it should be discussed.

Conclusion

Your study makes a significant contribution to the field by developing culturally appropriate physical activity promotion strategies for older Chinese adults in the UK. The integration of evidence, theory, and stakeholder perspectives is commendable. Addressing the points above will further enhance the clarity, rigor, and impact of your work.

I look forward to seeing the final version of your manuscript and wish you the best with your important research.

VERSION 1 - AUTHOR RESPONSE

**Reviewer: 1**<br>**Dr. Carl-Philipp Jansen, Heidelberg University**	
Comments to the Author:<br>Cultural adaptation: to really understand what is meant by this, clarification and definition is required. In the current stay, this is a very vague term and it is not clear what would fall under "culture".<br><br>The BCW was applied, helping to identify 27 appropriate BCTs for this purpose. The logic model helps to illustrate the relationships between identified barriers, intervention components, mechanisms of action, and expected outcomes. The subsequent think-aloud interviews were an important step in reaching an understanding of the intevention's potential in reaching its target.<br><br>The only practical strategies that actually addressed needs specific to Chinese older adults (opposed to the non-Chinese general population) were 7, 9, and 10 (translation); the other strategies would most probably be useful to all older adults. It seems this study came with a huge effort to simply come up with three rather intuitive solutions to "make the intervention more Chinese". I suggest to address this.	Thanks for this important comment. We agree that many intervention components may also be applicable to the wider older adult population, as participants experienced not only culture-related barriers, but also broader structural and ageing-related barriers commonly reported among older adults generally.<br><br>As suggested by Netto et al., 2010, "cultural adaptation" in this study was integrated within broader behavioural and contextual influences, rather than being limited to uniquely culture-specific components alone.<br><br>To clarify this, we have added a definition of "culturally appropriate" in the background section:<br><br>*"In this study, the term "culturally appropriate" refers to an intervention tailored to older Chinese adults in the UK by incorporating cultural, social, and contextual factors inffuencing PA behaviour, reffecting a holistic understanding of behaviour rather than treating culture as an isolated component [8]."*<br><br>In addition, we have added a relevant discussion point in the Discussion section to address this comment:<br>*"Importantly, "culturally appropriate" in this study does not refer solely to culture-specific components (e.g., language or cultural values). Instead, it reffects a broader, population-informed approach that incorporates structural factors (e.g.,*

	accessibility), social inffuences (e.g., peer support), and sense of belonging. This is supported by evidence generated across the intervention development process, including qualitative interviews with older Chinese adults in the UK, which consistently identified that PA behaviour is shaped by structural, contextual, cultural, and individual-level factors.<br>*This broader conceptualisation is also consistent with previous guidance on developing behaviour change interventions for ethnic minority groups [33-35], which emphasises integrating cultural relevance with wider social, environmental, and structural determinants to enhance engagement and accessibility. The intervention in this study incorporated culturally relevant materials, family and community involvement, and digital support to enhance engagement and address social and environmental barriers."*

Coming out of this theory-driven approach and person-centered efforts, the intervention went through an iteration of changes that helped to sharpen its components. It will be interesting to see the results of the feasibility testing.	Thank you for this comment. We agree with the reviewer's point regarding feasibility testing.<br><br>The feasibility study has been reported separately in a distinct manuscript and is therefore not included in the current paper.

Why are coercion and restriction deemed unacceptable and by whom?	Thank you for this important question. We agree this is an important point and the decision was made based on the suggestion of PPIE activity. We have added the following clarification to explain why these two approaches were deemed unacceptable.<br><br>*"while restriction and coercion were deemed unacceptable as they conffict with core principles of autonomy, dignity, and informed consent in healthcare and research, this position was also confirmed by the PPIE group, who emphasised the need for voluntary, respectful, and non-coercive participation."*
There is no clear reasoning why the Otago program was chosen. Is Otago appropriate? Is it ensured that this is the right fit for this population?	Thanks for the comment.<br><br>The Otago Exercise Programme was selected because it is an evidence-based exercise programme designed to improve strength and balance in older adults. It was also chosen for practical reasons, as the physiotherapist involved in the study could support intervention delivery. However, we also acknowledge that the Otago programme, as a relatively low-intensity exercise intervention, may not have been sufficiently challenging for some more active participants.<br><br>The following description has been added to the Findings section to provide the reasons for choosing the Otago Exercise Programme:

	"The Otago Exercise Programme was selected as an evidence-based strength and balance intervention, with practical delivery supported by a Chinese-speaking physiotherapist to enhance accessibility and acceptability for the target population."<br><br>In addition, we have added the limitation of choosing Otago in the discussion section:<br><br>*"A potential mismatch between participant characteristics and the intervention was identified, as the Otago Exercise Programme may not have been sufficiently challenging for more active individuals. Future research should therefore consider incorporating progressive exercise options and more ffexible, capacity-based approaches to better match participant ability levels."*
There are predominantly women for the intervention development (one man). Please discuss implications.	Thanks for the comment. A sentence has been added in the limitation section:<br><br>*"The predominance of female participants in the intervention development process may have inffuenced the findings and intervention preferences identified, potentially limiting the transferability of the intervention to older Chinese men."*

It is argued that people had limited access to smartphones for apps, but WeChat was kept in as part of the intervention despite having the same problem? Please explain.	Thanks for the comment. We agree that this is an important issue related to digital access and digital literacy. This intervention component was included to address participants' need for peer support, which was often limited by geographical dispersion and the inability to meet in person, especially among ethnic minority group. Considering variations in digital access and confidence, participants were given the option to use either WeChat or WhatsApp, both of which are widely used and familiar to many participants, as suggested by the PPIE group. The feasibility and acceptability of this digital component will be further examined in the subsequent feasibility study.<br><br>A sentence has been added in the discussion section to reflect this concern:<br><br>*"However, its delivery via digital platforms may be constrained by variability in participants' digital access and literacy, and therefore its feasibility and acceptability require further evaluation in the subsequent feasibility study."*
Singing is suggested but it is not PA; please discuss.	Thanks for the comment. We agree with this point.<br><br>Singing has been replaced with "group-based dancing and singing-based social activities" to better reflect its physical activity relevance and context within the study.

<table>
<tr><td colspan="2">Reviewer: 2
Dr. Arnaud Philippot, KU Leuven Comments to the Author:

Thank you for the opportunity to review your important and innovative work. I read your manuscript with great interest, particularly your integration of the Person-Based Approach (PBA) and the Behaviour Change Wheel (BCW) to address the unique needs of older Chinese adults in the UK. Combining these frameworks is a significant challenge, and I commend the authors for their thoughtful and inspiring approach. Below, I made some suggestions and questions aimed at further strengthening the clarity, rigor, and impact of your study. When there are several suggestion, I also choose minor revisions as I am confident the authors will address these points effectively.</td></tr>
<tr><td>Part 1.
• You mention the participation with older Chinese adults.

Could you please provide more detail on the methodology employed? For instance, was a systematic approach used for data collection and qualitaitve analysis? Were interviews audio-recorded, and were field notes taken? Clarifying these aspects would enhance the transparency and reproducibility of your methods.</td><td>Thanks for your comment and I am sorry for the confusion. This is also relevant to the following comments.

This study primarily followed a person-based approach, whereby the intervention was developed based on qualitative findings conducted at the preparatory stage, which has been reported in a separate paper. This paper focus on the intervention development and this process was led by the research team with input from the PPIE group, and no qualitative data were collected during the intervention design stage. The exception was the think-aloud interviews conducted during the intervention optimisation stage.

Although the PPIE group contributed throughout the development process, this was undertaken as an information-gathering and consultation approach rather than formal data collection.</td></tr>
</table>

	Details of data collection process during the think-aloud interview has been added:<br><br>*"Interviews were audio-recorded with participants' consent and conducted either face-to-face or online depending on participants' preferences. Field notes were also taken during the interviews."*
• Guiding Principles: The development of guiding principles is a key strength of your study. However, it is not entirely clear which elements were informed by the literature, previous qualitative studies, or direct input from the target population. Could you specify how participant input was integrated into the design objectives and intervention components? If qualitative analysis was conducted, a brief description of the process would be valuable.	Thanks for this comment. This study forms part of a PhD project, and prior to the intervention development process, we conducted qualitative interviews with 27 older Chinese adults to explore factors influencing their PA behaviour. This study has been reported separately and briefly findings are reported in the background section.<br><br>The guiding principles were then developed based on these qualitative findings in the earlier stage, with additional input from the PPIE group to prioritise the most commonly reported and important factors.<br><br>To improve clarity, the following description has been added to the Methods section where the guiding principles are introduced:<br><br>*"In this study, the guiding principles were informed by prior qualitative interviews with 27 older Chinese adults during the preparatory stage and refined with the PPIE group to ensure they reffected the most important determinants of PA behaviour."*

• Representation of Participant Voices: To ensure the voices of participants are accurately represented, did you use a specific model or framework for qualitative analysis? If so, please consider including this information.	Thank you for the comment. The findings from the qualitative interviews with 27 participants have been reported elsewhere, and the relevant reference has been provided in this paper. Data were analysed using thematic analysis. The analysis initially followed an inductive approach, allowing themes to emerge from the data without reference to a predefined theoretical framework. At a later stage, the identified themes were mapped onto the COM-B framework using a deductive approach to facilitate interpretation of the findings within a behavioural science perspective.<br><br>For the think-aloud interviews, the following details have been added to the Data Analysis section. We would be grateful for your view on whether this is sufficient.<br><br>*"A rapid, iterative approach specifically recommended for think-aloud interviews was followed, with feedback tabulated to capture positive and negative comments, barriers, and suggested improvements [2c]. The coding framework suggested by the PBA is presented in Appendix S. The MoSCoW prioritisation criteria (Must have, Should have, Could have, Would like) were applied to guide modification decisions. Proposed changes were discussed with the PCIE group and research team to reach a consensus."*

Table 1: Barriers and Facilitators<br>• Safety Concerns: Under "lack of motivation," the phrase "build confidence in managing safety concerns" could be clarified. For example, does this refer to fears of falling, moving in public, or other specific concerns? A brief elaboration would help readers understand the context.	Thank you for this comment. Yes, safety concerns included fears of falling during walking or exercise, as well as concerns about worsening existing conditions such as hypertension or heart disease, which led some participants to feel less confident about engaging in physical activity. We have now added these examples after "Safety Concerns" to improve clarity.<br><br>*"safety concerns such as concerns about falling or concerns related to conditions such as hypertension or heart disease,"*
Results<br>APEASE Criteria<br>Selection of Intervention Functions and BCTs: The use of APEASE criteria for selecting intervention functions and BCTs is well-justified. However, it would be helpful to explain the specific criteria used to exclude certain components. Including this information in the supplementary data would maximize transparency and allow readers to fully appreciate the decision-making process.	Thank you for this comment. The tables outlining the APEASE criteria and the selection/exclusion of intervention functions and BCTs have now been added to the Appendix 5 and 6 to improve transparency.<br><br>Appendix 5 Applying APEASE to Select Intervention Functions<br><br>Appendix 6 Identifying BCTs Using Evidence and APEASE
Delivery Model<br>Step 8 of BCW It's not clear if at the stage you have included the participation of the participants, could you clearly indicated when you included voices in this study of older Chinese participants living in UK?<br>Largely, could you indicate in each step of CBW if the opinions of your participants have been included or it was based on literature with research expert analysis.	Thank you for this comment. For the overall project, participant involvement (older Chinese adults) occurred during the preparatory (semi-structured interviews (n=27), published separately) and optimisation stages (think-aloud interviews (n=10)).<br><br>The subsequent intervention development stages, including the guiding principles, intervention planning table, behavioural analysis, selection of

	intervention functions and BCTs, and delivery model, were primarily researcher-led and informed by the qualitative findings. However, these stages were also refined through ongoing input from the PCIE group to ensure relevance, acceptability, and cultural appropriateness.<br><br>We apologise for any confusion and confirm that this clarification has now been added to the manuscript.<br><br>*"This process was led by the lead researcher, guided by the guiding principles, with regular discussions with the research team and PCIE group:"*
Part 2 :<br>Participatory Health Research<br>Terminology: Consider using the term "participatory health research" instead of or in addition to "patient-centered approach," as your study actively involved participants in the research process. This would more accurately reflect the collaborative nature of your work in the research process.	Thank you very much for this helpful comment. After careful consideration, and in line with our study design, we are not fully confident that the term "participatory health research" accurately reflects our methodology. Although the study involved stakeholder (PCIE) input throughout the intervention development process, the research was primarily led by the research team and grounded in qualitative findings collected during the preparatory stage.<br><br>As outlined in the Taxonomy of Approaches to Intervention Development proposed by O'Cathain et al. (2019), approaches such as co-production, co-creation, co-design, and cooperative design are classified within the *Partnership* category, which is defined as an approach in which

	the people whom the intervention is intended to benefit are involved in decision-making throughout the development process and have at least equal decision-making power to members of the research team.<br>In contrast, the approach adopted in our study was the Person-Based Approach (PBA) (Yardley et al., 2015), which falls within the *Target Population-Centred* category. Although the intervention development process was informed by extensive input from the target population and stakeholders, decision-making responsibility ultimately remained with the research team.<br><br>Therefore, we have retained the term *Person-Based Approach*, as it more accurately reflects the methodological approach and study design used in this research.
Traditionally, qualitative research recommends a sample size of 12 participants for qualitative interviews to ensure methodological rigor and data saturation. While I acknowledge that budgetary constraints may have limited participant recruitment, the final sample of ten individuals could be perceived as slightly underpowered. To further strengthen the methodological justification and be more aligned with the participatory research I encourage the authors to cite relevant literature supporting the adequacy of this sample size in participatory research required ; 9-12 participants, corresponding to your study with the inclusion of 10 participants. For example, recent studies in this field have successfully employed similar sample sizes, as demonstrated in:<br>https://doi.org/10.1016/j.mhpa.2026.100758<br>https://formative.jmir.org/2024/1/e51693	Thank you very much for your comment. In contrast to traditional qualitative research, which aims to develop an in-depth and comprehensive understanding of a phenomenon, the **think-aloud interviews** in this study were specifically designed to identify participants' immediate responses, usability issues, and views on intervention components. Evidence suggests that a relatively small sample (minimum of approximately five participants) is sufficient to identify the majority of usability and intervention-related issues.

	The following clarification has been added to the sample size section of the think-aloud interviews. We would be grateful for your view on whether this is sufficient.<br><br>*"It has been suggested that five think-aloud interviews with end-users can identify the majority of usability issues [25]. This think-aloud study aimed to recruit a minimum of 5–10 participants, considering the financial and time constraints of the research project."*
Workshop Details: Could you provide more information on how the workshops were conducted? For example, what activities were included, and how were they structured to engage participants?	Thank you very much for this comment. We apologise for the confusion.<br>The workshop to deliver PA information and the Otago exercises was part of the intervention itself rather than the intervention development process. Therefore, no workshop activities were conducted as part of the intervention development stage reported in this paper.
Clarification of PBA and BCW Integration: If audio recordings and qualitative analysis were used to inform the selection of BCTs, consider including this information in Stage 1 to highlight the participatory nature of your approach.	As mentioned earlier, the intervention was developed using the PBA and BCW, informed by findings from earlier interviews with the target population. The selection of intervention functions and BCTs was primarily researcher-led, with support and feedback from the PPIE group. However, no additional qualitative data collection was conducted during this specific stage of the intervention development process.<br><br>We have rewritten this section to better describe this process:

	"In addition to identifying behavioural targets for change, the BCW outlines an eight-step process for intervention development, of which seven were applied in this project [15]. This process was led by the lead researcher, guided by the guiding principles, with regular discussions with the research team and PCIE group. "
Cultural Appropriateness<br><br>Your introduction effectively highlights the importance of culturally appropriate interventions. In the discussion, consider further emphasizing how your intervention's cultural components (e.g., booklets, workshops, social media groups) address the specific needs of older Chinese adults. Comparing your findings with similar studies would provide valuable context and support for your conclusions, such as:<br><br>https://link.springer.com/article/10.1007/s10903-024-01587-5<br><br>https://doi.org/10.1016/j.jad.2025.04.128	Thank you for the comment. We agree that it is important to further clarify what "culturally appropriate" means in this study.<br><br>The following description has been added to the Discussion section to better reflect the culturally appropriate nature of the intervention:<br><br>*"Importantly, "culturally appropriate" in this study does not refer solely to culture-specific components (e.g., language or cultural values). Instead, it reffects a broader, population-informed approach that incorporates structural factors (e.g., accessibility), social inffuences (e.g., peer support), and sense of belonging. This is supported by evidence generated across the intervention development process, including qualitative interviews with older Chinese adults in the UK, which consistently identified that PA behaviour is shaped by structural, contextual, cultural, and individual-level factors.*<br>*This broader conceptualisation is also consistent with previous guidance on developing behaviour change interventions for ethnic minority groups [33-35],*

	which emphasises integrating cultural relevance with wider social, environmental, and structural determinants to enhance engagement and accessibility. The intervention in this study incorporated culturally relevant materials, family and community involvement, and digital support to enhance engagement and address social and environmental barriers"
Policy Categories (BCW Step 6), while the focus on community- and individual-level strategies is understandable, the exclusion of policy-level actions in step 6 due to resource constraints is noted. Could you discuss how future research might address this gap, perhaps by exploring partnerships with policymakers or community organizations to enhance the scalability and sustainability of your intervention?	Thank you very much for this important comment. The following description has been added to the Future Work section:<br><br>*"In addition, as this study did not include BCW Step c policy-level strategies, future research should consider engaging local policymakers and community organisations to support wider implementation, scalability, and long-term sustainability of the intervention beyond individual- and community-level delivery."*
To ensure the sustained and scalable impact of your intervention, it is essential to clarify which stakeholders will be responsible for its ongoing delivery, implementation, and updates. Could the authors elaborate on the following points?<br>• Identification of Stakeholders: Who are the key individuals or organizations envisioned to lead and maintain the intervention?<br>• Engagement Strategies: How do you plan to engage these stakeholders and participants in the long term? Are there existing partnerships or networks that could facilitate their involvement?<br>• Funding and Resources: What mechanisms or funding sources will support the continued operation of the intervention?<br>If not, it should be discussed.	Thank you for the comment.<br><br>The following description has been added in the "further work" in the discussion section:<br><br>*Future implementation may involve collaboration with community organisations, Chinese community groups, and local health or social care services supporting older adults in the UK. Ongoing engagement through existing community networks and the PCIE group may help maintain relevance and acceptability, while future*

	scalability and sustainability may require support from public health initiatives, NHS/community partnerships, or future implementation funding.
Conclusion<br>Your study makes a significant contribution to the field by developing culturally appropriate physical activity promotion strategies for older Chinese adults in the UK. The integration of evidence, theory, and stakeholder perspectives is commendable. Addressing the points above will further enhance the clarity, rigor, and impact of your work.<br>I look forward to seeing the final version of your manuscript and wish you the best with your important research.	Thank you very much for your positive and encouraging feedback. We appreciate your recognition of the study's contribution and the integration of evidence, theory, and stakeholder perspectives.<br>We are also grateful for your constructive comments, which have helped improve the clarity, rigour, and quality of the manuscript. Please let us know if any changes are needed.

Reviewer: 1
If you have selected 'Yes' above, please provide details of any competing interests.: Not applicable

Reviewer: 2
If you have selected 'Yes' above, please provide details of any competing interests.: not applicable

---

VERSION 2 - REVIEW

**Reviewer**	**1**
**Name**	**Jansen, Carl-Philipp**
**Affiliation**	**Heidelberg University, Geriatric Center**
**Date**	**19-Jun-2026**
**COI**	

I congratulate the authors to their work; all my comments have been addressed appropriately which leaves me with nothing further to comment.

**Reviewer**	**2**
**Name**	**Philippot, Arnaud**

Affiliation **KU Leuven, Research Group for Adapted Physical Activity and Psychomotor Rehabilitation**

Date **07-Jul-2026**

COI

Thank you very much for your thoughtful response and the careful revisions you have made.

I have only one remaining comment. Regarding the addition below concerning the interviews conducted, could you provide a reference to this previous study? If it is currently in preparation, this could potentially be accepted to be referenced like this to the Editor's agreement.

"In this study, the guiding principles were informed by prior qualitative interviews with 27 older Chinese adults during the preparatory stage and refined with the PCIE group to ensure they reflected the most important determinants of PA behaviour."

VERSION 2 - AUTHOR RESPONSE

**Reviewer: 2**<br>**Dr. Arnaud Philippot, KU Leuven**	
I have only one remaining comment. Regarding the addition below concerning the interviews conducted, could you provide a reference to this previous study? If it is currently in preparation, this could potentially be accepted to be referenced like this to the Editor's agreement.<br><br>"In this study, the guiding principles were informed by prior qualitative interviews with 27 older Chinese adults during the preparatory stage and refined with the PCIE group to ensure they reflected the most important determinants of PA behaviour.	Thank you for your helpful comment. The qualitative interview study has now been accepted for publication and is currently in press in *BMC Geriatrics*.<br><br>We have therefore added the corresponding reference to this section to support the statement.<br><br>*"In this study, the guiding principles were informed by prior qualitative interviews with 27 older Chinese adults during the preparatory stage [11] and refined with the PCIE group to ensure they reffected the most important determinants of PA behaviour."*
