# Supplementary material for: Developing culturally appropriate physical activity promotion strategies for older Chinese adults in the UK: an evidence-based, theory-based and person-based intervention development study
Source: BMJ Open. 2026 Sep 3;16(9):e117339. doi: 10.1136/bmjopen-2026-117339 (PMC13548221; doi:10.1136/bmjopen-2026-117339)
Supplement: Supplementary file 2 [file bmjopen-16-9-s001.docx]

### **Appendix 1 GUIDED Checklist for Intervention Development**

| **GUIDED Item** | **Description in this study** | **Page/Section** |
| --- | --- | --- |
| **1. Context** | Older Chinese adults in the UK face cultural, linguistic, and structural barriers to engaging in PA, with limited culturally appropriate interventions available. | Introduction |
| **2. Purpose** | To develop culturally appropriate PA promotion strategies for older Chinese adults in the UK. | Introduction |
| **3. Target population** | Community-dwelling older Chinese adults living in the UK. | Methods – Participants |
| **4. Use of theory** | The Behaviour Change Wheel (BCW) and COM-B model informed identification of behavioural determinants and intervention functions; the Person-Based Approach (PBA) guided user-centred design. | Methods – Intervention development |
| **5. Use of evidence** | Two systematic reviews and a qualitative interview study informed intervention content, delivery modes, and cultural tailoring. | Methods; Previous studies |
| **6. Development approach** | Integrated MRC framework, PBA, and seven steps of the BCW across design and optimisation stages. | Methods – Study design |
| **7. Stakeholder contribution** | A PCIE group contributed through material review and think-aloud interviews. | Methods – PCIE |
| **8. Decision-making** | Design decisions were informed by evidence, theory, stakeholder feedback, and research team consensus. | Methods – Development process |
| **9. Changes during development** | Based on think-aloud interviews, three components were retained, one added, and three removed; others were modified. | Results – Optimisation |
| **10. Uncertainties** | Feasibility, acceptability, and effectiveness remain to be tested. | Discussion |
| **11. Intervention description** | Final intervention includes culturally tailored PA booklets, monthly workshops, and social media support groups. | Results – Final intervention and TIDieR appendix |
| **12. Mode of delivery** | Community-based workshops, printed materials, and digital social media support. | Methods – Intervention |
| **13. Resources & feasibility** | Designed for delivery in community settings using low-cost materials and existing platforms. | Discussion |
| **14. Future evaluation** | A feasibility RCT is planned to assess acceptability, adherence, and preliminary effectiveness. | Discussion |

### **Appendix 2 Definition of the problem in behavioural terms**

| What is the behaviour? | Insufficient PA, defined as not meeting WHO guidelines for aerobic, strength, and balance activities. |
| --- | --- |
| Where does the behaviour occur? | Any location where PA can take place, such as a home, community centre, or park. |
| Who is involved in performing the behaviour? | Older Chinese adults in the UK who do not meet the PA guidelines. |

### **Appendix 3 The description of the target behaviour**

| Target behaviour | Increase PA with aerobic, strength, and balance activities at levels suited to participants’ circumstances, with the ultimate goal of progressing toward WHO guidelines for older adults. |
| --- | --- |
| Who needs to perform the behaviour? | Older Chinese adults in the UK who currently do not meet the recommended PA guidelines. |
| What do they need to do differently to achieve the desired change? | They need to engage more in aerobic, strength, and balance activities to progress toward the WHO PA guidelines. |
| When do they need to do it? | Any time that is suitable for them. |
| How often do they need to do it? | Frequently, such as 30 minutes of aerobic activity daily and strength and balance exercises two times per week. |
| With whom do they need to do it? | Engaging in PA alone or with others. |

### **Appendix 4** **Using the COM-B model to identify what needs to change**

| **COM B** | **Factor** | **What needs to happen for the target behaviour to occur** | **Need for change** |
| --- | --- | --- | --- |
| Physical Capability | Health conditions | Provide suitable physical activities for older adults with health conditions (e.g. urinary incontinence). | Yes – provide appropriate PA |
|  | Skills | Older adults need specific skills to perform activities such as balance and strength exercises. | Yes – provide training in relevant PA |
| Psychological Capability | Knowledge | Older adults need to understand (1) the importance of PA and (2) how to choose appropriate activities. | Yes – increase knowledge of PA and age-appropriate recommendations |
|  | Language | Older Chinese adults in the UK may require PA information and programmes in Chinese. | Yes – provide PA information and programmes in Chinese |
|  | Behaviour regulation | Older adults need the ability to monitor their PA (e.g. set goals and track progress). | Yes – support self monitoring and goal setting |
|  | Experience | Previous experience with PA may encourage participation. | No – not targeted for change |
| Physical Opportunity | Physical environment | Access to safe and convenient spaces (e.g. parks) supports PA engagement. | No – not feasible to change; provide information instead |
|  | PA programmes | Provide low cost, safe, and easy to follow PA programmes. | Yes – offer suitable PA programmes for older Chinese adults |
|  | Information resources | Ensure access to information about PA and existing programmes or groups. | Yes – provide relevant information on PA options |
|  | Technology | Use tools such as WeChat and pedometers to support PA. | Yes – use WeChat for support and pedometers for self monitoring |
|  | Pet ownership | Pet ownership may encourage daily PA. | No – not modifiable within this project |
|  | Weather and season | Weather and season influence PA participation. | No – provide alternative options for different conditions |
| Social Opportunity | PA group or company | Exercising with a group or buddy promotes engagement. | Yes – introduce groups and encourage buddy or virtual support |
|  | Social support | Support from family, friends, and the community helps maintain PA. | Yes – involve family and foster peer support |
|  | Time for PA | Some older adults have limited time due to responsibilities. | Yes – support integration of PA into daily routines |
|  | Traditional Chinese culture | Including traditional elements increases relevance and motivation. | Yes – provide culturally tailored PA options |
|  | Influence of COVID 19 | COVID 19 disrupted regular activities. | No – post-pandemic period |
| Reflective Motivation | Belief in benefits of PA | Believing that PA benefits outweigh risks supports engagement. | Yes – provide education on PA benefits |
|  | Family harmony | Belief that PA contributes to family harmony. | Yes – highlight this benefit |
|  | Fear of falls | Fear of falling is a major barrier to PA. | Yes – provide safe PA options and fall prevention guidance |
|  | Goal setting | Clear daily or weekly targets support participation. | Yes – assist with goal setting |
| Automatic Motivation | Perceived benefits | Noticing benefits reinforces continued participation. | Yes – support tracking and reflection |
|  | Emotion | Positive emotions and enjoyment motivate PA. | Yes – encourage recognition of emotional benefits |
|  | Socialisation | Opportunities for social interaction promote motivation. | Yes – facilitate social aspects of PA |
|  | Habit or routine | Incorporating PA into daily routines supports adherence. | Yes – encourage habit formation |
|  | Passing time or fulfilment | Some engage in PA to pass time or for fulfilment. | No – situational factor |

### **Appendix 5 Applying APEASE to Select Intervention Functions**

| Population group based on COM-B | Barrier to target behaviour | Intervention component | Example | Does the BCT meet the APEASE criteria |
| --- | --- | --- | --- | --- |
| Lack of physical capability | Health strength/  Lack of skills | Training | Using demonstration, feedback, and practice to improve physical or psychological skills such as the Capability to analyse information and plan based on that information | Yes |
|  |  | Enablement | Providing physical or social support or material or financial resources that make it possible or easier to enact a behaviour | Yes |
| Lack of psychological capability | Knowledge/  Language barrier | Education | Informing or explaining things to increase knowledge or understanding relating to a behaviour | Yes |
|  |  | Training | Using demonstration, feedback, and practice to improve physical or psychological skills such as ability to analyse information and plan on the basis of that information | Yes |
|  |  | Enablement | Providing physical or social support, or material or financial resources that make it possible or easier to enact a behaviour | Yes. |
| Lack of physical opportunity | Lack of suitable environment/ programme/ information resources | Training | Using demonstration, feedback, and practice to improve physical or psychological skills such as ability to analyse information and plan on the basis of that information | Yes |
|  |  | Restriction | Using formal social rules to set boundaries for a behaviour | Not practicable. There is no option to restrict in this context. |
|  |  | Environmental restructuring | Shaping the physical or social world inhabited by a person to make a behaviour easier or harder, or appear more or less normal, or to add or remove prompts | No |
|  |  | Enablement | Providing physical or social support, or material or financial resources that make it possible or easier to enact a behaviour | Yes |
| Lack of social opportunity | Lack of company/social support/Time | Restriction | Using formal social rules to set boundaries for a behaviour | No |
|  |  | Environmental restructuring | Shaping the physical or social world inhabited by a person to make a behaviour easier or harder, or appear more or less normal, or to add or remove prompts | No |
|  |  | Modelling | Providing an example for people to imitate, learn from or aspire to | Yes |
|  |  | Enablement | Providing physical or social support, or material or financial resources that make it possible or easier to enact a behaviour | Yes |
| Lack of automatic motivation | Prefer other activity rather than PA | Persuasion | Using words and images to get people to feel liking or disliking for something in order to influence behaviour | Yes |
|  |  | Enablement | Providing physical or social support, or material or financial resources that make it possible or easier to enact a behaviour | Yes |
|  |  | Incentivisation | Applying rewards to a behaviour | Yes |
|  |  | Coercion | Applying costs or punishments to a behaviour | Not acceptable to older adults |
|  |  | Training | Using demonstration, feedback, and practice to improve physical or psychological skills such as ability to analyse information and plan based on that information | Yes |
|  |  | Environmental restricting | Shaping the physical or social world inhabited by a person to make a behaviour easier or harder, or appear more or less normal, or to add or remove prompts | No |
|  |  | Modelling | Providing an example for people to imitate, learn from or aspire to | Yes |
| Lack of reflective motivation | Believe that the harmfulness of PA outweighs the benefit | Education | Informing or explaining things to increase knowledge or understanding relating to a behaviour | Yes |
|  |  | Persuasion | Using words and images to get people to feel liking or disliking for something to influence behaviour | Yes |
|  |  | Incentivisation | Applying rewards to a behaviour | Yes |
|  |  | Coercion | Applying costs or punishments to a behaviour | No. Not acceptable to the older adults |
|  |  | Modelling | Providing an example for people to imitate, learn from or aspire to | Yes |

APEASE criteria (Affordability, Practicability, Effectiveness and cost-effectiveness, Acceptability, Side effects or safety, and Equity)

### **Appendix 6 Identifying BCTs Using Evidence and APEASE**

| **BCT Categories** | **BCTs** | **From Evidence** | **APEASE Criteria** |
| --- | --- | --- | --- |
| **1. Goals and planning** | 1.1 Goal setting (behaviour) | Yes | Yes – Set PA targets based on guidelines and current PA level |
|  | 1.2 Problem solving | Yes | Yes – Identify COM-B barriers and provide support strategies |
|  | 1.4 Action planning | Yes | Yes – Develop weekly PA plans and record activities |
|  | 1.5 Review behaviour goal(s) | No | Added – Review weekly goals |
|  | 1.6 Discrepancy between current behaviour and goal | No | Added – Adjust targets for gaps |
|  | 1.9 Commitment | Yes | No – Not formalised |
| **2. Feedback and monitoring** | 2.1 Monitoring of behaviour by others without feedback | Yes | No – Feedback always given |
|  | 2.2 Feedback on behaviour | Yes | Yes – Physiotherapist feedback during sessions |
|  | 2.3 Self-monitoring of behaviour | Yes | Yes – Logbook to record PA |
|  | 2.4 Self-monitoring of outcome(s) | Yes | Yes – Record strength, balance, mood |
|  | 2.5 Monitoring of outcome(s) without feedback | Yes | No – Feedback included |
|  | 2.6 Biofeedback | Yes | No – No devices used |
|  | 2.7 Feedback on outcomes | Yes | No – Not reviewed individually |
| **3. Social support** | 3.1 Social support (unspecified) | Yes | Yes – Identify peer buddy |
|  | 3.2 Social support (practical) | Yes | Yes – Partner assists participation |
|  | 3.3 Social support (emotional) | Yes | Yes – Peer encouragement & motivational messages |
| **4. Shaping knowledge** | 4.1 Instruction on how to perform behaviour | Yes | Yes – Chinese booklet & physiotherapist |
| **5. Natural consequences** | 5.1 Information about health consequences | Yes | Yes – Explain health benefits of PA |
|  | 5.3 Information about social & environmental consequences | Yes | Yes – Explain social benefits |
|  | 5.4 Monitoring of emotional consequences | No | Added – Record emotional responses |
|  | 5.6 Information about emotional consequences | Yes | Yes – Explain mood/wellbeing benefits |
| **6. Comparison of behaviour** | 6.1 Demonstration of the behaviour | Yes | Yes – Physiotherapist demonstrates exercises |
|  | 6.2 Social comparison | Yes | Yes – Share experiences in workshops & social media |
|  | 6.3 Information about others’ approval | Yes | No – Not emphasised |
| **7. Associations** | 7.1 Prompts/cues | Yes | Yes – Booklet visible & reminders sent |
| **8. Repetition and substitution** | 8.1 Behavioural practice/rehearsal | Yes | Yes – Practice in workshops & home |
|  | 8.3 Habit formation | Yes | Yes – Exercise in same daily context |
|  | 8.7 Graded tasks | Yes | No – No structured progression |
| **9. Comparison of outcomes** | 9.1 Credible source | Yes | Yes – NHS/GOV.UK/physiotherapist |
|  | 9.2 Pros and cons | Yes | No – Not formally discussed |
| **10. Reward and threat** | 10.2 Material reward (behaviour) | Yes | No – No tangible incentives |
|  | 10.4 Social reward | Yes | Yes – Praise for goals achieved |
|  | 10.8 Incentive (outcome) | Yes | No – No outcome-based incentive |
|  | 10.9 Self-reward | Yes | No – Not instructed |
|  | 10.11 Future punishment | Yes | No – Punitive framing avoided |
| **11. Regulation** | 11.2 Reduce negative emotions | Yes | No – No formal emotion training |
| **12. Antecedents** | 12.1 Restructuring physical environment | Yes | No – Environment unchanged |
|  | 12.2 Restructuring social environment | Yes | Yes – Workshops & social media facilitate interaction |
|  | 12.5 Adding objects to the environment | Yes | Yes – Provide equipment (resistance bands) |
| **13. Identity** | 13.1 Identification of self as role model | Yes | No – Not targeted |
| **15. Self-belief** | 15.1 Verbal persuasion about capability | Yes | Yes – Encouraging messages |
|  | 15.3 Focus on past success | Yes | No – Past success not reviewed |
| **16. Covert learning** | 16.3 Vicarious consequences | No | Added – Share peer stories & achievements |

### **Appendix 7 The identified BCTs and delivery model**

| **No.** | **BCTs** | **Intervention component** | **Delivery model** |
| --- | --- | --- | --- |
| 1 | 1.1. Goal setting (behaviour) | Set PA targets based on guidelines and current PA level. | Booklet |
| 2 | 1.2. Problem-solving | Identify COM-B barriers and provide support strategies. | Booklet |
| 3 | 1.4. Action planning | Make weekly PA plans and record activities in a logbook. | Booklet |
| 4 | 1.5. Review behaviour goal(s) | Review weekly PA goals and set new ones. | Booklet |
| 5 | 1.6. Discrepancy between current behaviour and goal | Review discrepancies between PA levels and goals, then adjust targets. | Booklet |
| 6 | 2.2. Feedback on behaviour | Physiotherapist provides feedback during Otago sessions. | Workshop |
| 7 | 2.3. Self-monitoring of behaviour | Use a logbook to record and monitor daily PA. | Booklet |
| 8 | 2.4. Self-monitoring of outcomes of behaviour | Self-monitor outcomes (e.g., strength, balance, mood) in the logbook. | Booklet |
| 9 | 3.1. Social support (unspecified) | Encourage participants to identify a buddy for peer support. | Booklet; Workshop; Social media group |
| 10 | 3.2. Social support (practical) | Find a support partner for practical help. | Booklet; Workshop; Social media group |
| 11 | 3.3. Social support (emotional) | Receive peer support and motivational messages from the researcher. | Workshop; Social media group |
| 12 | 4.1. Instruction on how to perform a behaviour | Provide a Chinese version of the Otago booklet; Chinese-speaking physiotherapist leads exercises. | Booklet; Workshop |
| 13 | 5.1. Information about health consequences | Provide information about the health consequences of PA. | Booklet; Workshop |
| 14 | 5.3. Information about social and environmental consequences | Provide information about the social consequences of PA (e.g., socialisation). | Booklet; Workshop |
| 15 | 5.4. Monitoring of emotional consequences | Record emotional consequences of PA. | Booklet; Workshop |
| 16 | 5.6. Information about emotional consequences | Provide information about the emotional benefits of PA (e.g., mood). | Booklet; Workshop |
| 17 | 6.1. Demonstration of the behaviour | Physiotherapist demonstrates Otago exercises at workshops. | Workshop |
| 18 | 6.2. Social comparison | Share PA experiences in workshops and social media groups. | Workshop; Social media group |
| 19 | 7.1. Prompts/cues | Keep the exercise booklet visible and send reminders of upcoming exercise events. | Workshop; Social media group |
| 20 | 8.1. Behavioural practice and rehearsal | Practise Otago exercises in workshops and at home. | Booklet; Workshop |
| 21 | 8.3. Habit formation | Exercise in the same context repeatedly (e.g., after dinner). | Booklet; Social media group |
| 22 | 9.1. Credible source | Use trusted sources (NHS, GOV.UK, Age UK); Otago delivered by a physiotherapist. | Booklet; Workshop |
| 23 | 10.4. Social reward | Congratulate participants for achieving targets. | Social media group |
| 24 | 12.2. Restructuring the social environment | Workshops and social media groups create opportunities to connect and share PA. | Workshop; Social media group |
| 25 | 12.5. Adding objects to the environment | Provide small equipment (e.g., resistance bands). | Workshop |
| 26 | 15.1. Verbal persuasion about capability | Send encouraging messages to boost confidence in PA. | Booklet; Workshop; Social media group |
| 27 | 16.3. Vicarious consequences | Share peer PA stories and participants’ own achievements. | Booklet; Workshop; Social media group |

### **Appendix 8 Topic guide for think-aloud interviews**

(Aimed at exploring participants’ views about the developed physical activity promotion strategies)

- **Topic 1: Opinions on culturally appropriate physical activity strategies**
  Participants will be asked questions such as:
  - “What do you think about these physical activity promotion strategies?”
  - “What do you think about <insert strategy feature>?”
- **Topic 2: Suggestions for intervention improvement**
  Participants will be asked questions such as:
  - “Which parts of the programme do you like best or least?”
  - “How do you think each strategy could be improved?”

### **Appendix 9 Coding framework suggested by the PBA for think-aloud interviews**

| Code | Stands For | Means |
| --- | --- | --- |
| IMP | Important for behaviour change | Important change. For example, it may be important because it is likely to impact engagement or behaviour change, or for practical, ethical or safety reasons. |
| EAS | Easy and uncontroversial | An easy and feasible change that doesn’t involve any major design changes. For example, a participant was unsure of a technical term, so you add a definition. |
| REP | Repeatedly | This was said repeatedly, by more than one participant. |
| EXP | Experience | This is supported by experience. Please specify what kind of experience, for example: PPI, Experts, Literature |
| NCON | Does not contradict | This does not contradict experience (e.g. evidence), or the Logic Model, or the Guiding Principles |

### **Appendix 10 Characteristics of the study sample (n = 10)**

| **Variable** | **Total** |
| --- | --- |
| Age | 68.6 (5.5) |
| Years in the UK | 27.4 (18.4) |
| Gender |  |
| Female | 9 (90.0%) |
| Male | 1 (10.0%) |
| Marital status |  |
| Married | 10 (100.0%) |
| First language |  |
| Cantonese | 6 (60.0%) |
| Mandarin | 4 (40.0%) |
| Education |  |
| Junior high school | 2 (20.0%) |
| High school | 4 (40.0%) |
| College/University or higher | 4 (40.0%) |
| PA frequency per week |  |
| <1 time | 2 (20.0%) |
| 2–3 times | 3 (30.0%) |
| >4 times | 5 (50.0%) |

### **Appendix 11 An overview of the initial culturally appropriate PA**

- **Core Booklet.** A culturally tailored, nine-page PA promotion booklet

Page 1 – Health Benefits of PA: Explains the health benefits of PA, as well as other benefits mentioned in previous interviews, such as opportunities for social interaction.

Page 2 – PA Recommendations: Summarises PA recommended based on the WHO PA guideline

Page 3 – Target Setting: Encourages participants to set PA targets based on the PA recommendation and their current PA levels.

Page 4 – Problem Identification and Solutions: Utilises COM-B model questions to help identify individual barriers to PA and suggest strategies to overcome them.

Page 5 – Tips to Integrate PA into Daily Life: Offers practical advice on incorporating PA into daily routines.

Page 6 – Safety Tips: Provides guidance on exercising safely.

Page 7 – Information on Local PA Programmes: Lists accessible and culturally relevant local PA opportunities.

Page 8 – Story Pages: Features relatable stories to model behaviour change and boost motivation.

Page 9 – Additional Support: Outlines further resources and contact points for ongoing encouragement and assistance.

Page 10 – Strength and Balance Exercise Booklet. The strength and balance training exercises from the NHS website were translated into Chinese.

**PA Logbook**. A logbook for tracking PA routines and progress over time, aiming to enhance self-monitoring and accountability.

- **Material Incentives**. Offering financial incentives to increase motivation for PA
- **Telephone Support**. Providing regular telephone call to encourage PA
- **WeChat Group**. An WeChat group was created to provide virtual opportunity for peer support
- **Exercise App**. Proposed use of NHS-endorsed PA apps. Removed due to limited smartphone access and digital literacy among participants.
- **Fitness Tracker**. Wearable devices to assist participants in monitoring their daily activity levels, helping to increase awareness and promote behaviour change.

### **Appendix 12** **Changes to intervention elements**

| ***Intervention page*** | ***Negative Comments*** | ***Positive Comments*** | ***Possible Change*** | ***Reason for change*** | ***MoSCoW*** | ***Implementation stage*** |
| --- | --- | --- | --- | --- | --- | --- |
| **Education** booklet Page 1-Benefit of PA | "This image is not very clear. I understand that you want to say that PA can reduce these diseases, but the description is a bit confusing. It’s better to keep it simple.” (P 9) | "Usually, people only talk about the health benefits of exercise, so it's nice to see you including other factors, like meeting people. Honestly, that’s even more important than the health benefits!  You see, we’ve just turned 60, and health isn’t a big issue yet. For them, exercising is more about socialising and having fun!” (P10)  “Traditional Chinese medicine is truly profound, especially ‘The Yellow Emperor’s Inner Classic—it contains a lot of really useful knowledge. Since you're developing something with Chinese characteristics, it's important to include elements of TCM. You can explain why PA is important from a TCM perspective.”(P 7) | - Replace the health benefits image with simple sentences.  - The benefits from the Traditional Chinese Medicine perspective are retained as originally presented.  - Other benefits remain the same, including opportunities to meet people, maintain independence and avoid becoming a burden, stay happy, manage weight, and look good. | EAS | Could have | Changed |
| Education booklet Page 2-WHO PA guideline | "There’s too much to read. I agree this knowledge is useful, but I don’t think older people will want to read all of this—it’s just too much... I think it would be better to add some pictures, like people doing Tai Chi. These words are too boring for older people.” (P9)  "I think some of these activities, like golf, may not be very popular among the Chinese population. Many of us like Tai Chi and singing. I think you should add these activities." (P1) | "I think the information here is useful. Usually, I just walk and think that's enough, but this page taught me things I didn’t know before." (P4) | - Replace the text description with the image from the UK Chief Medical Officers' PA Guidelines (2019). - Add traditional Chinese exercises, such as Tai Chi and singing | EAS  REP | Must have | Changed |
| Education booklet Page 3-Smart goal | "I don’t like the feeling of others setting targets for me. I prefer to decide what to do on my own. If I want to go for a walk, then I do. I think people should be able to set their own target based on their own situation" (P1)   "Some of the information on this page is kind of similar to the earlier one—“How much PA should I do?” I don’t think you need to repeat it again here."(P4)  "To be honest, this page is a bit too complex for me." (P5) | “Having a plan is really helpful, especially for me. I just had surgery, so I made one—starting with 5 minutes and slowly increasing to 30 minutes. I think it's a great idea!”(P2)  “It will be useful to write down the PA target, make it more formal” (P9) | - Simplify the target-setting table and merge it with the problem identification page."  - Allow participants to write down their current PA level and their target level. | IMP  REP | Must have | Changed |
| Education booklet Page 4-Identify problem | "I think it's best to make these questions simpler so they are easier to understand” (P8). | "It is a good idea to think about different situations, because everyone is different” (P4) | - The COM-B–based questions were redesigned using simple and short sentences to help identify problems and guide users to appropriate support resources.  - The “identify problem” was then combined with the goal-setting page. | EAS  NCON | Could have | Changed |
| Education booklet Page 5-Tips to integrate PA into daily life | “Some of the tips are saying the same things” (P9) | I think this information is really helpful—like getting off the bus one stop early. I’d actually like to try that! (P10) | - Tips have been rewritten to make them simple and clear. | EAS  NCON | Could have | Changed |
| Education booklet Page 6-Safety tips | No negative comment | “This page looks good, and the information on it is useful for helping people stay safe.” (P8) | No change was made to this page | NC | NC | NC |
| Education booklet Page 7-PA Information Resources | "I think this idea’s great, but it doesn’t cover everything. I actually know a few other exercise programs too, and I can send the information to you" (P3) | “The information on this page is really helpful. I’ve been in Manchester for many years, but I didn’t know there is a Chinese walking group!” (P7) | More PA programs were added. | EAS  NCON | Could have | Changed |
| Education booklet Page 8-Story of how PA benefits others | No negative comment | "I really like this page. I can read other people’s stories and feel inspired."(P10) | No change was made to this page | NC | NC | NC |
| Education booklet Page 9-Additional support | No negative comment | “I never thought I needed to talk about my exercise with my GP. I usually only contact them when I’m not very well, so I think this information could be a good reminder for people.” (P9) | No change was made to this page | NC |  | NC |
| Exercise booklet | “The words are too small to read. Why not use fonts that suit older Asian women? You know, they’re the ones most likely to read this booklet.” (P9)  It’s nice to see you translate it into Chinese, but to be honest, I don’t think it can really make a difference. I bet they’ll take it home but won’t look at it. If you can find a professor to teach them, that might work.” (P6) | “You know, many older people don’t know how to do these exercises, like strength and balance, so they just walk. I think it would be helpful, but it would be even better if someone could teach them face to face.” (P7) | A larger font size was used, but due to time and resource constraints, the character images were not changed.  A group exercise session was added to supplement the intervention. A Chinese physiotherapist will be invited to teach participants strength and balance exercises. | IMP  EAS  REP | Must have | Changed |
| Exercise Logbook | “It’s just too complicated. Keep it simple — I can just write down what exercise I did each day” (P10)  “Keep it simple — instead of writing, just let them make a mark. Don’t ask them to write, because some older adults don’t even know how to write.” (P6) | “It’s like a diary, right? You can look back and see how much you’ve done, and that could be motivating.” (P6) | The logbook has been re-designed to make it simple to use. | IMP  REP | Must have | Changed |
| Workshop-Presentation based on the booklet | No negative comment | Yeah, it’s better if you give a talk about the benefits and everything in the booklet. It’s a really good idea—because you know, they might not read it at all if you just hand them the booklet. (P8) | A newly added element based on the first round think-aloud interview | IMP  REP  EXP (PCIE) | Should have | Changed |
| Workshop-Group discussion | No negative comment | “The seniors will love this—just getting together, chatting, maybe having some cake! You know, when they hear others are doing lots of exercise, they’ll be like, “Oh, maybe I should start too.” That might actually work better than just talking to them about it” (P10) | A newly added element based on the first round think-aloud interview | IMP  REP  EXP (PCIE) | Should have | Changed |
| Workshop-Otago exercises led by a Chinese-speaking physiotherapist | No negative comment | “I think it would be helpful, but it would be even better if someone could teach them face to face.” (P7)”  “That’s a great idea. If you can find someone to teach them, I will definitely come to learn” (P7) | A newly added element based on the first round think-aloud interview | IMP  REP  EXP (PCIE) | Should have | Changed |
| Social media support group | I don’t think they’ll like to download and learn to use a new app. We know people from mainland China all use apps, but we (Cantonese) don’t use that. We use WhatsApp.” (P2)  “Yes, why don’t you give people the option to use the app they prefer?” (P5)  In addition, participants suggested that in-person gatherings would be more effective than virtual-only groups: “That would be useful if you can gather people together, just like today—buy some cake, they will love to come. You can then give a talk or show them how to do exercises.” (P5) | Sure, for example, we also have a group chat for our hiking activities to send out notices. If you send encouraging messages every day, it can still be somewhat helpful when they see them. (P1) | Participants will be given the option to use either WeChat or WhatsApp of their choice. | IMP  EAS  REP  EXP (PCIE) | Must have | Changed |
| PA monitor | “I think it’s just useless. The text is too small, older people don’t know how to charge it, and you need to connect it to a phone to see things—older people just don’t know how to use it. It’s very unlikely they’ll record their steps every day.” (P2) | “I check how many steps I’ve walked every day, and I make sure to reach a certain number. It really motivates me. If I haven’t walked enough, I’ll walk around at home until I hit my goal.” (P6) | Provide an age-friendly pedometer with a large display, memory function, and one that doesn’t need frequent charging. | IMP  EAS  REP  EXP (PCIE) | Must have | Changed |
| Incentive | "Giving rewards might work for kids. But for older people, especially those who have money, it's hard to change them. They just don't care." (P1)  “Even they start to come for the money, but I feel it will not last” (P5) | “For some older adults, they would be happy to come if you gave them some money or a small gift” (P3) | Element removed | IMP  EXP (PCIE) | Should have | Changed |
| Telephone support. | Calling people isn’t a great idea. I used to be part of a program where I had to call every day and ask how much they walked. It was tiring, and often no one answered the phone." (P1) | I think it’ll work if you give them a call and remind them—they’ll do it. But if you don’t, they probably won’t move at all...(P5) | Element removed | EXP (PCIE) | Should have | Changed |
| NHS-recommended PA management app  (Couch to 5k, Active 10) | “They (Apps) are usually too complicated. We’re older, not so good with this kind of stuff—especially when it’s all in English. There’s just no way for us to use it. It won’t work.” (P2)  “Yes, some older adults, like my aunts, never use it, even though their children bought them smartphones. (P7). | No positive comments | Element removed | IMP  EAS  REP | Should have | Changed |

Note: IMP: Important for behaviour change; EAS: Easy and uncontroversial (i.e., An easy and feasible change that doesn’t involve any major design changes); REP: Repeatedly(i.e., This was said repeatedly, by more than one participant); EXP: Experience (i.e., This is supported by experience); NCON: Does not contradict (i.e., This does not contradict experience (e.g. evidence), or the Logic Model, or the Guiding Principles); NC: Not changed. The MoSCoW method categorises components as Must have, Should have, Could have, or Won’t have

### **Appendix 13 TIDieR Description of the Intervention**

| **TIDieR Item** | **Description** |
| --- | --- |
| **1. Brief name** | Culturally appropriate physical activity (PA) promotion strategies for older Chinese adults in the UK. |
| **2. Why (rationale, theory, goals)** | Older Chinese adults in the UK are more likely to be physically inactive and face culturally specific barriers. These challenges align with the COM-B domains of capability, opportunity, and motivation. This intervention aims to promote PA using the Behaviour Change Wheel, combined with the Person-Based Approach, with cultural tailoring to enhance relevance and acceptability. |
| **3. What (materials)** | **Booklets:** (1) A culturally appropriate core PA booklet, with each page designed to enhance capability, opportunity, and motivation; (2) Chinese Otago home exercise booklet; (3) PA logbook. All materials are available in Traditional and Simplified Chinese.  **Digital support:** WeChat or WhatsApp group.  **Workshops:** Three monthly in-person workshops.  **Equipment:** Resistance band and pedometer. |
| **4. What (procedures)** | Participants receive the booklets at baseline and attend three workshops over 12 weeks. They are asked to complete home-based Otago strength and balance exercises at least twice per week, record their PA in the logbook, and monitor steps using a pedometer. They also engage in a social media support group, where they receive motivational and reminder messages from the researcher and are encouraged to share their PA experiences to provide peer support. |
| **5. Who provided** | Workshops are delivered by the lead researcher, with a qualified Chinese-speaking physiotherapist demonstrating Otago exercises. The social media groups are facilitated by the researcher, and all participants are encouraged to share their PA behaviours and interact within the group. |
| **6. How (mode of delivery)** | Printed materials, face-to-face group workshops, and online peer support via WeChat or WhatsApp. |
| **7. Where** | Workshops are delivered at the university; online support is provided via WeChat or WhatsApp. |
| **8. When and how much** | Duration: 12 weeks. Workshops: three sessions (every 4 weeks), approximately 90 minutes each. Home exercises are ongoing, with participants encouraged to achieve at least 150 minutes of PA per week and to complete the Otago exercises twice per week. Three motivational messages are sent via social media by the researcher. |
| **9. Tailoring** | The intervention was developed based on the needs and cultural preferences of older Chinese adults. Content includes traditional Chinese values, local Chinese PA programme information, and a translated Otago exercise booklet. Participants may choose Traditional or Simplified Chinese materials and either WeChat or WhatsApp. PA goals and activities are self-selected and recorded in the logbook. |
| **10. Modifications** | Based on think-aloud interviews, three components were retained, one was added, three were removed, and others were modified to improve clarity, usability, and cultural relevance. The intervention may be further modified based on the findings of the feasibility RCT. |
| **11. How well (planned fidelity)** | A fidelity checklist was prepared. Standardised materials and delivery guides were used, and workshop attendance, logbooks, pedometer data, and social media engagement were monitored. |
| **12. How well (actual fidelity)** | To be assessed and reported in the feasibility evaluation. |
